# Synthesis, spectral analysis, and DFT studies of the novel pyrano[3,2-*c*] quinoline-based 1,3,4-thiadiazole for enhanced solar cell performance

**DOI:** 10.1016/j.heliyon.2024.e39468

**Published:** 2024-10-17

**Authors:** Ibtisam Alali, Magdy A. Ibrahim, N. Roushdy, Al-Shimaa Badran, Alaa Muqbil Alsirhani, A.A.M. Farag

**Affiliations:** a Department of Chemistry, College of Science, Jouf University, Sakaka, Aljouf ,72341, Saudi Arabia; b Chemistry Department, Faculty of Education, Ain Shams University, Roxy, Cairo, 11711, Egypt; c Electronics Materials Dep. Advanced Technology& New Materials Research Inst, City of Scientific Research & Technological Applications (SRTA-City), New Borg El-Arab City, Alexandria, 21934, Egypt; d Thin-film Laboratory, Physics Department, Faculty of Education, Ain Shams University, Roxy, Cairo, 11711, Egypt

**Keywords:** Pyrano[3,2-*c*]quinoline, Thiadiazole, Density functional theory, Nonlinear optical, Single oscillator model, Photovoltaic performance

## Abstract

In this study, we synthesized a novel compound, 3-(5-amino-1,3,4-thiadiazol-2-yl)-6-ethyl-4-hydroxy-2*H*-pyrano[3,2-*c*]quinoline-2,5(6*H*)-dione (**ATEHPQ**), through a condensation reaction between 6-ethyl-4-hydroxy-2,5-dioxo-5,6-dihydro-2H-pyrano [3,2-c]quinoline-3-carboxaldehyde and thiosemicarbazide, followed by oxidative cyclization. We characterized **ATEHPQ** using elemental analysis, IR, ^1^H and ^13^C NMR spectroscopy, and mass spectrometry. Density Functional Theory (DFT) calculations with the B3LYP/6–311++G(d,p) basis set were employed to optimize the molecular geometry and analyze global reactivity descriptors, including HOMO-LUMO energies. The Molecular Electrostatic Potential (MEP) map was used to identify reactive sites, and drug-likeness studies indicated potential pharmaceutical applications. Notably, **ATEHPQ** showed a higher first hyperpolarizability (β_tot_) compared to urea, suggesting its suitability for nonlinear optical applications. We also determined the Miller indices for **ATEHPQ**'s preferred orientations using a specialized program. Williamson–Hall analysis revealed an average crystal size of 26.08 nm and a lattice strain of 6.3 × 10^−3^. The thin films exhibited three distinct absorption peaks at 2.8, 3.41, and 4.21 eV, with a direct energy gap of 2.43 eV. Dispersion parameters from the single oscillator model provided oscillator and dispersion energies of 3.12 eV and 14.21 eV, respectively, with a high-frequency dielectric constant of 4.71. The **ATEHPQ** thin films, when combined with n-Si, demonstrated significant improvements in photovoltaic performance: the open-circuit voltage (V_oc_) rose from 0.13 V to 0.521 V, the short-circuit current (Isc) increased from 0.253 mA to 2.94 mA, the fill factor (FF) improved from 0.238 to 0.33, and the efficiency (η) grew from 0.71 % to 4.64 % with increased illumination intensity. These results highlight the excellent photovoltaic and photodetection capabilities of **ATEHPQ** thin films, underscoring their potential for advanced optoelectronic and solar cell applications.

## Highlights

• Synthesized ATEHPQ via oxidative cyclization of thiosemicarbazone in boiling ethanol.
• ATEHPQ structure confirmed through IR, NMR, mass spectrometry, and elemental analysis.
• DFT optimization revealed molecular geometry and reactivity descriptors of ATEHPQ.
• Identified reactive sites of ATEHPQ using Molecular Electrostatic Potential (MEP) map.
• ATEHPQ showed higher first hyperpolarizability, suitable for nonlinear optical applications.
• Thin films exhibited absorption peaks at 2.8, 3.41, and 4.21 eV, with a 2.43 eV energy gap.
• ATEHPQ thin films showed enhanced photovoltaic performance and photodetection.
• Improved solar cell efficiency from 0.71 % to 4.64 % under 100 mW/cm^2^ illumination.

## Graphical abstract

**Figure:**
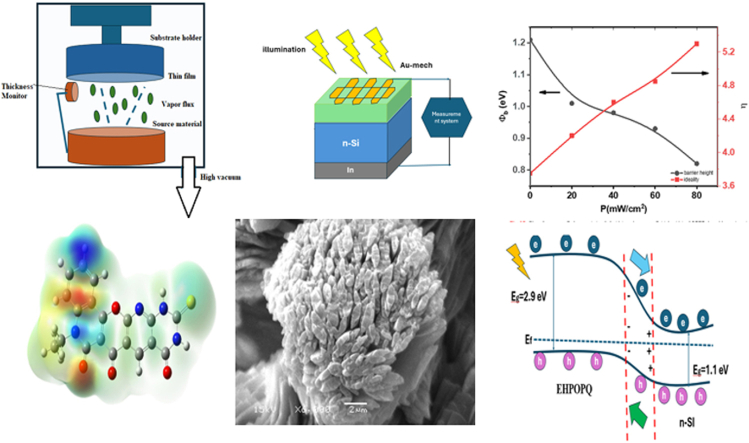

## 1 Introduction

Organic semiconductors, composed primarily of carbon-based materials, have emerged as a promising class of materials for electronic and optoelectronic applications. Unlike their inorganic counterparts, organic semiconductors offer advantages such as low-cost processing, flexibility, and potential for large-area devices. Their unique properties, arising from the delocalized π-electron systems, enable applications in organic field-effect transistors (OFETs), organic light-emitting diodes (OLEDs), and organic photovoltaic (OPV) cells. A subset of organic semiconductors, heterocyclic compounds incorporate atoms other than carbon and hydrogen into their cyclic structures. These heteroatoms, such as nitrogen, oxygen, and sulfur, significantly influence the electronic properties of the materials. Heterocyclic semiconductors have gained considerable attention due to their potential for tuning optoelectronic properties through structural modifications. These compounds have found applications in various fields, including organic electronics, photonics, and sensing [[1], [2], [3], [4], [5]].

Pyrano [3,2-c]quinoline derivatives represent an intriguing class of heterocyclic compounds with a fused pyran and quinoline core structure. The presence of nitrogen and oxygen heteroatoms within their molecular framework contributes to their potential for exhibiting interesting electronic and optical properties. Previous studies have explored their applications in diverse areas, including pharmaceuticals and materials science. However, their potential as organic semiconductors, particularly in the context of solar cell applications, remains largely untapped [[6], [7], [8], [9], [10], [11], [12]].

The optical properties of pyrano [3,2-c]quinoline derivatives have been investigated to some extent, with studies focusing on their absorption and emission characteristics. However, a comprehensive understanding of their optoelectronic properties, including charge carrier mobility and exciton diffusion length, is still lacking. Regarding solar cell applications, there is limited literature on the utilization of pyrano [3,2-c]quinoline derivatives as active materials in photovoltaic devices. While some studies have explored the photovoltaic properties of related compounds, the specific potential of pyrano[3,2-*c*]quinolines in this domain remains largely unexplored. Pyrano [3,2-c]quinoline derivatives have emerged as a focal point of interest within the realm of heterocyclic chemistry due to their versatile structural framework and potential for diverse applications. These compounds, characterized by the fusion of pyran and quinoline rings, have demonstrated promise in various fields, including pharmaceuticals and materials science [13,14].

Previous studies [[15], [16], [17]] have highlighted the ability of pyrano [3,2-c]quinoline derivatives to address a range of biological challenges, alongside their notable optical properties and insights gained from computational analyses using density functional theory (DFT). Expanding on these findings, this research represents a pioneering effort to investigate, for the first time, the structural, optical, and optoelectronic properties of a new pyrano [3,2-c]quinoline derivative, **ATEHPQ**. This study not only examines the compound in its powdered form but also explores its thin-film applications, particularly focusing on its potential use in solar cells.

By combining the expertise of our research team in organic synthesis, spectroscopy, and materials science, we seek to establish comprehensive structure-property relationships for **ATEHPQ**. This collaborative approach will enable us to optimize the synthesis process, characterize the compound in detail, and evaluate its potential for application in solar cell technology. Through a systematic investigation, we aim to contribute significantly to the advancement of pyrano [3,2-c]quinoline-based materials and their integration into functional devices.

This study represents a synergistic effort to expand the knowledge base of one of the pyrano [3,2-c]quinoline derivatives (**ATEHPQ**) and to explore their untapped potential in the field of organic electronics. This study introduces the synthesis of a novel compound, 3-(5-amino-1,3,4-thiadiazol-2-yl)-6-ethyl-4-hydroxy-2H-pyrano [3,2-c]quinoline-2,5(6H)-dione (**ATEHPQ**), derived from the condensation reaction of 6-ethyl-4-hydroxy-2,5-dioxo-5,6-dihydro-2H-pyrano [3,2-c]quinoline-3-carboxaldehyde with thiosemicarbazide followed by oxidative cyclization. The structure of **ATEHPQ** was confirmed through elemental analysis and various spectral techniques, including IR, ^1^H and ^13^C NMR, and mass spectrometry. The molecular geometry of **ATEHPQ** was optimized using Density Functional Theory (DFT) with the B3LYP/6–311++G(d,p) basis set, revealing detailed insights into global reactivity descriptors and HOMO-LUMO energies. The reactive sites were mapped using the Molecular Electrostatic Potential (MEP) method. Additionally, ^1^H and ^13^C NMR chemical shifts were computed with the Gauge-Independent Atomic Orbital (GIAO) method.

This work is novel in its detailed investigation of the nonlinear optical (NLO) properties of **ATEHPQ**, showing its high first hyperpolarizability (βtot) compared to standard urea, highlighting its potential for NLO applications. For the first time, the Miller indices for preferred orientations of **ATEHPQ** were calculated using a specific program, and the average crystal size and lattice strain were determined from Williamson–Hall plots. The thin films of **ATEHPQ** exhibited significant optical characteristics, including distinct absorption peaks and a direct energy gap. The current work is also highlighted focusing on the structural, optical, and electronic properties of **ATEHPQ**. The research combines experimental techniques with computational modeling to assess the compound's suitability for advanced material applications. The study particularly emphasizes the photovoltaic performance of **ATEHPQ** thin films in heterojunctions with n-Si, showcasing notable improvements in open-circuit voltage, short-circuit current, fill factor, and efficiency under varying illumination intensities. This investigation aims to contribute to the development of enhanced optoelectronic devices and solar cells, demonstrating the potential of **ATEHPQ** for advanced applications in these fields.

## 2 Experimental details

Melting points were recorded using a Stuart SMP3 digital apparatus. Infrared spectra were acquired with an FTIR Nicolet IS10 spectrophotometer and a PerkinElmer 293 spectrophotometer, employing KBr pellets. Nuclear magnetic resonance (NMR) spectra were obtained with a Mercury-300BB spectrometer, where ^1^H NMR (300 MHz) and ^13^C NMR (75 MHz) were recorded in DMSO-d6, with tetramethylsilane (TMS) as the internal standard. Mass spectrometry was performed using a GC-2010 Shimadzu gas chromatography mass spectrometer (70 eV). Elemental analyses were conducted using a PerkinElmer CHN-2400 analyzer.

### 2.1 Synthesis of 3-(5-amino-1,3,4-thiadiazol-2-yl)-6-ethyl-4-hydroxy-2H-pyrano [3,2-c]quinoline-2,5(6H)-dione (3)

Step 1 To a solution of carboxaldehyde **5** (0.57 g, 2 mmol) in absolute ethanol (15 mL), thiosemicarbazide (0.18 g, 2 mmol) in distilled water (5 mL) was added. The reaction mixture was heated under reflux for 30 min. The pale-yellow crystals obtained during heating were filtered and crystallized from AcOH/H_2_O, mp 240–241 °C, yield (0.59 g, 82 %).
Step 2 A mixture of thiosemicarbazone **2** (0.72 g, 2 mmol) and ferric chloride (0.64 g, 4 mmol) in aqueous dioxane (80 %, 40 mL) was heated under reflux for 6 h. The reaction mixture was poured onto a 10 % aqueous sodium carbonate solution (50 mL) and stirred for 2 h. The solid so formed was filtered, washed several times with water, and crystallized from DMF/H_2_O to give compound **3** as yellow crystals, mp > 300 °C, yield (0.53 g, 74 %). IR (KBr, cm^−1^): 3402 (OH), 3304, 3215 (NH_2_), 3043 (CH_arom._), 2979, 2928 (CH_aliph._), 1720 (C=O_α–pyrone_), 1656 (C=O_quinoline_), 1604 (C=N), 1575 (C=C). ^1^H NMR (300 MHz, DMSO-*d*_6_, *δ*): 1.26 (t, 3H, *J* = 6.9 Hz, CH_3_), 4.05 (q, 2H, *J* = 6.9 Hz, CH_2_), 7.28 (t, 1H, *J* = 7.2 Hz, H-9), 7.56 (d, 1H, *J* = 7.2 Hz, H-7), 7.81 (t, 1H, *J* = 7.2 Hz, H-8), 8.07 (d, 1H, *J* = 7.2 Hz, H-10), 8.86 (s, 2H, NH_2_ exchangeable with D_2_O), 11.54 (s, 1H, OH exchangeable with D_2_O). ^13^C NMR (75 MHz, DMSO, *δ*): 12.7 (CH_3_), 36.6 (CH_2_), 101.7 (C-3), 106.8 (C-4a), 125.2 (C-10a), 126.9 (C-9), 128.4 (C-7), 129.3 (C-8), 130.5 (C-10), 132.3 (C-6a), 137.9 (C-3′_thiadiazole_), 143.4 (C-5′_thiadiazole_), 149.4 (C-4), 153.6 (C-4b), 163.8 (C-2 as C=O_α–pyrone_), 168.4 (C-5 as C=O_quinoline_). Mass spectrum (*m*/*z*, *I* %): 356 (69), 328 (100), 300 (41), 272 (22), 257 (36), 229 (24), 201 (30), 189 (52), 175 (16), 161 (19), 133 (42), 119 (18), 105 (39), 91 (28), 77 (46) and 64 (27). Anal. Calcd for C_16_H_12_N_4_O_4_S (356.36): C, 53.93; H, 3.39; N, 15.72; S, 9.00 %. Found: C, 53.72; H, 3.21; N, 15.61; S, 8.87 %.

### 2.2 Computational details

The molecular structure was determined using the density functional theory (DFT)/B3LYP/6–311++ G(d,p) basis set [[18], [19], [20], [21]]. Every computation was carried out using the Gaussian 09 program [22]. The DFT/B3LYP/6–311++G(d,p) level of theory was chosen because it provides a reliable balance between computational cost and accuracy for investigating the structural, electronic, and optical properties of organic molecules. The B3LYP functional is widely used in quantum chemical studies due to its ability to accurately predict molecular geometries and electronic properties. The 6–311++G(d,p) basis set was selected because it includes diffuse and polarization functions, which are essential for obtaining accurate results in systems involving non-covalent interactions, charge distributions, and excited states [[23], [24], [25]], making it particularly suitable for the detailed study of the **ATEHPQ** derivative in this work. The Gauss View 5.0 visualization application was used to extract the calculation results, display the optimized structures, and pinpoint the frontier molecular orbitals (FMOs). The Gauss view in optimized files was used to graphically present data on the molecular electrostatic potential (MEP) surface, highest occupied molecular orbital (HOMO), and lowest unoccupied molecular orbital (LUMO) [26]. The ^1^H and ^13^C NMR isotropic shielding were calculated using the GIAO method. The dipole moment (μ), mean polarizability (α), and first hyperpolarizability (β_tot_) were determined using DFT theory.

### 2.3 Preparation and characterization of ATEHPQ thin films

**ATEHPQ** thin films were deposited using a thermal evaporation technique in a high-vacuum chamber, with substrates carefully pre-cleaned to ensure optimal film quality. The thickness of **ATEHPQ** thin films was measured using a quartz crystal thickness monitor, which determined the thickness to be 300 nm. This method offers real-time monitoring and high precision during deposition. The microstructure of the deposited films was examined using a JEOL-JSM-636 OLA scanning electron microscope (SEM). The optical characteristics of the films, including light absorption and band gap measurements, were assessed with a UV–Vis spectrophotometer (JASCO-670 model).

### 2.4 Optical constants determination of ATEHPQ thin films

To determine the optical constants of **ATEHPQ** films, transmittance (T) and reflectance (R) measurements were carried out across a wide wavelength range from 190 to 2500 nm. These measurements were performed using a JASCO V-670 UV–Vis–NIR spectrophotometer, renowned for its accuracy in spectral analysis. The experimental transmittance (T_exp_) and reflectance (R_exp_) values obtained are essential for calculating the optical constants, including the refractive index (n) and the absorption index (k) of the **ATEHPQ** films.

### 2.5 Photovoltaic characteristics of ATEHPQ films/n-Si heterojunction

For evaluating the photovoltaic performance, hybrid heterostructures incorporating **ATEHPQ** films were fabricated. Current-voltage (I-V) measurements were performed under dark conditions and varying light intensities using a high-impedance source-measure unit, specifically the Keithley 2635A apparatus. This thorough methodology enabled a detailed investigation of the films' morphology, optical properties, and photovoltaic performance.

## 3 Results and discussion

### 3.1 Synthesis and molecular structure characterization

**Scheme 1:**
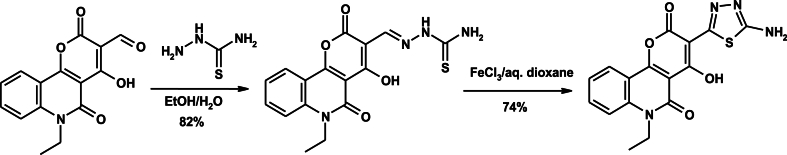
Formation of the novel **ATEHPQ, 3**.

**Scheme 2:**
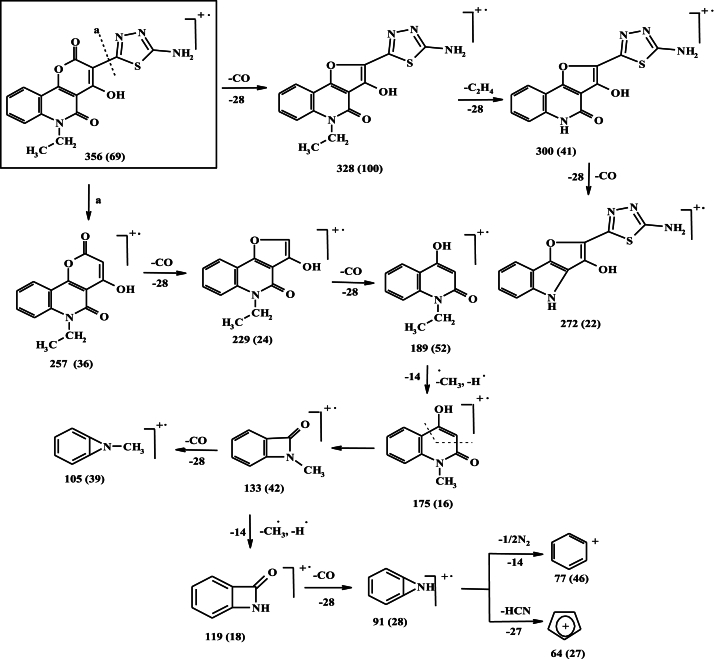
The mass fragmentation patterns of **ATEHPQ.**

In the present work, the reaction of 6-ethyl-4-hydroxy-2,5-dioxo-5,6-dihydro-2H-pyrano [3,2-c]quinoline-3-carbaldehyde (**1**) with thiosemicarbazide in boiling ethanol afforded the corresponding thiosemicarbazone **2** which upon oxidative cyclization, using ferric chloride in aqueous dioxane, yielded 3-(5-amino-1,3,4-thiadiazol-2-yl)-6-ethyl-4-hydroxy-2*H*-pyrano[3,2-*c*]quinoline-2,5(6*H*)-dione (**ATEHPQ, 3**) (Scheme 1) [27]. The IR spectrum of **ATEHPQ** (Fig. S1) showed characteristic absorption bands at 3402 (OH), 3304, 3215 (NH_2_), 1720 (C=O_α–pyrone_), 1656 (C=O_quinoline_) and 1604 cm^−1^ (C=N). The ^1^H NMR spectrum of **ATEHPQ** (Fig. S3) presented aromatic protons as typical triplet, doublet, triplet, and doublet signals at *δ* 7.28, 7.56, 7.81, and 8.07 ppm. The triplet and quartet signal attributable to ethyl protons were seen at *δ* 1.26 and 4.05 ppm, respectively. The amino and hydroxyl protons were recorded as D_2_O exchangeable signals at *δ* 8.86 and 11.54 ppm, respectively [28]. Also, the ^13^C NMR spectrum (Fig. S4) supported the identity of structure and presented 16 signals corresponding to the carbon atoms that constitute the scaffold of **ATEHPQ**. The upfield signals at *δ* 12.7 and 36.6 ppm were assigned to CH_3_ and CH_2_ carbons, respectively. Meanwhile, the downfield signals at 163.8 and 168.4 ppm were assigned to (C-2 as C=O_α–pyrone_) and (C-5 as C=O_quinoline_). The C-3 and C-5 of thiadiazole moiety were observed at *δ* 137.9 and 143.4 ppm, respectively. The structure of **ATEHPQ** was further confirmed using mass spectrum (Fig. S2) which presented the parent ion peak at *m/z* 356 which coincides with the suggested molecular weight 356.36 (C_16_H_12_N_4_O_4_S). The mass fragmentation patterns of **ATEHPQ** were depicted in Scheme 2.

### 3.2 Chemical reactivity parameters (global reactivity descriptors)

**Fig. 1:**
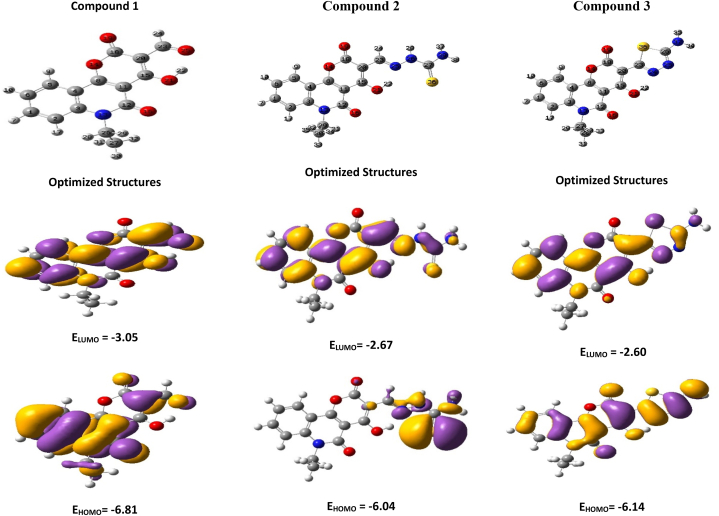
Molecular modeling depicting the electron density of the highest occupied molecular orbital (HOMO) and lowest unoccupied molecular orbital (LUMO) for compounds **1** to **3**.

Frontier molecular orbitals (FMOs) play an important part in molecular interactions by employing the electron donor-acceptor process. The highest occupied molecular orbitals (HOMOs) and lowest unoccupied molecular orbitals (LUMOs) are very important. The pictorial representation of HOMO and LUMO compositions of the current compounds is depicted in Fig. 1. HOMO and LUMO energies determine many chemical reactivity parameters like HOMO-LUMO energy gap (E_gap_), ionization potential (IP), electron affinity (EA), electronegativity (χ), global hardness (η), electrophilicity index (ω), and chemical potential (Pi) [28,29] using DFT/B3LYP/6–311++G(d,p) basis set (Table 1). HOMO-LUMO analysis provides information about the chemical hardness and softness of the molecule. Particularly, a greater E_gap_ is suggestive of a harder molecule, while a lower energy gap implies a softer molecular entity [30,31]. Thus, compound **1** has a considerably greater E_gap_ of 3.76 eV, indicating stronger stability and chemical hardness. However, compound **2** is softer than the other compounds. The soft molecule has higher polarizability and reactivity. Furthermore, the compound's negative chemical potential (Pi) indicates that it does not spontaneously break down into primal elements [32,33]. The chemical potential of the compounds is in the range of −4.36 to −4.93 eV.

**Table 1:** Global reactivity descriptors of the studied compounds **1**–**3**.

Parameters	Aldehyde 1	Compound 2	Product ATEHPQ (3)
E_HOMO_	−6.81	−6.04	−6.14
E_LUMO_	−3.05	−2.67	−2.60
IP (eV)	6.81	6.04	6.14
EA (eV)	3.05	2.67	2.60
E_gap_ (eV)	3.76	3.37	3.54
η (eV)	1.88	1.68	1.77
Pi (eV)	−4.93	−4.36	−4.37
ω (eV)	6.45	5.64	6.45
S **(eV**^**−**^**^1^)**	0.53	0.59	0.57
χ	4.93	4.36	4.37
ΔN_max_	2.62	2.59	2.47

In addition, higher electronegativity indicates a stronger electrophile [34]. The computed electronegativity value of 4.93 eV suggests that compound **1** is an electronegative molecule.A greater electrophilicity index (ω) indicates a chemical entity's ability to receive electrons, implying a more pronounced electrophilic nature [35,36]. Compound **1** has a higher electrophilicity index (ω) of 6.45.

### 3.3 Molecular electrostatic potential

**Fig. 2:**
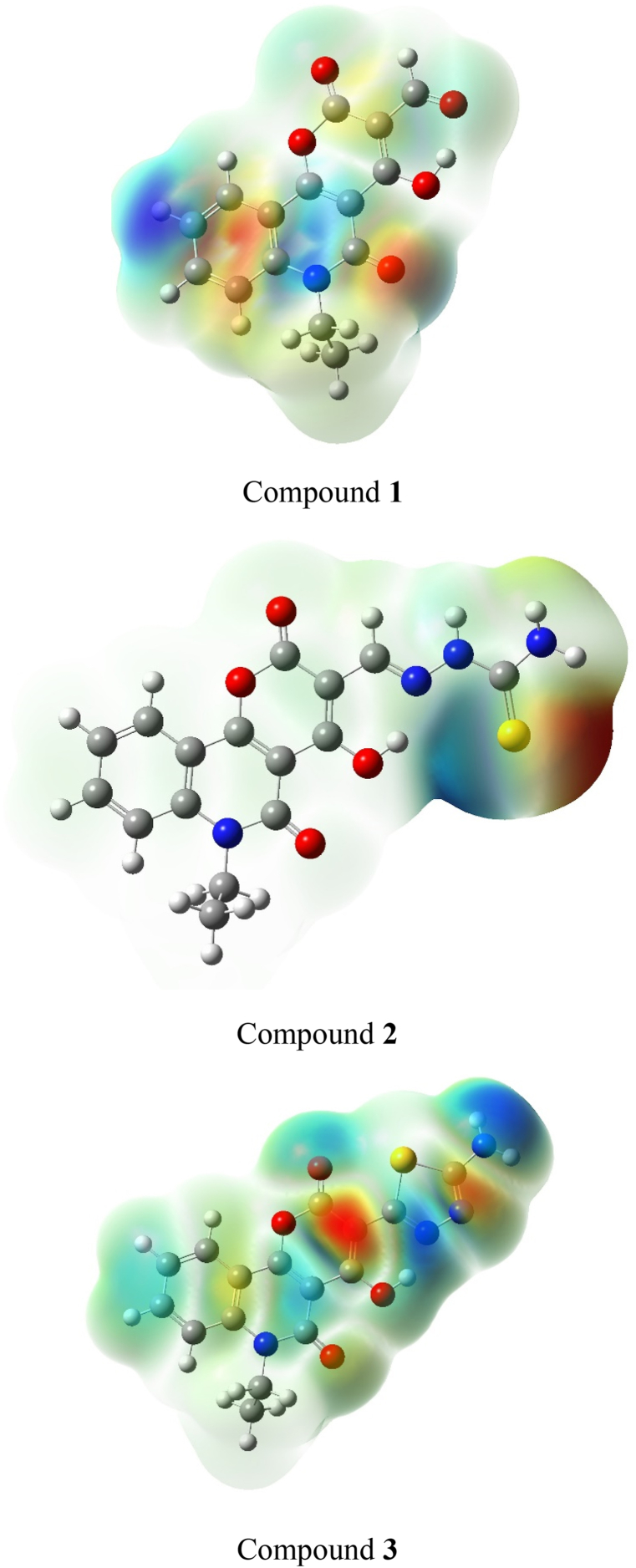
Molecular electrostatic potential for compounds 1 to 3.

MEP maps were developed to better understand the chemical reactivity of compounds. Using the B3LYP/6–311++G (d,p) method, Fig. 2 shows the MEP surface map of the prepared compounds. These maps indicate zones of positive and negative potential, which correlate to the overall distribution of electron density [37]. MEP maps depict potential variations using colors based on the relationship between electrostatic potential and total electron density, with red representing negative values and blue representing positive. MEP maps show blue areas with substantial electron attraction, indicating electrophilic reactivity, and red areas with significant electron repulsion, indicating nucleophilic reactivity. Green surfaces indicate that the potential is zero. The oxygen atoms have negative potential regions, whereas the hydrogen atoms have positive potential areas, indicating electrophilic and nucleophilic attack.

### 3.4 ^1^H NMR and ^13^C NMR spectroscopy

**Fig. 3:**
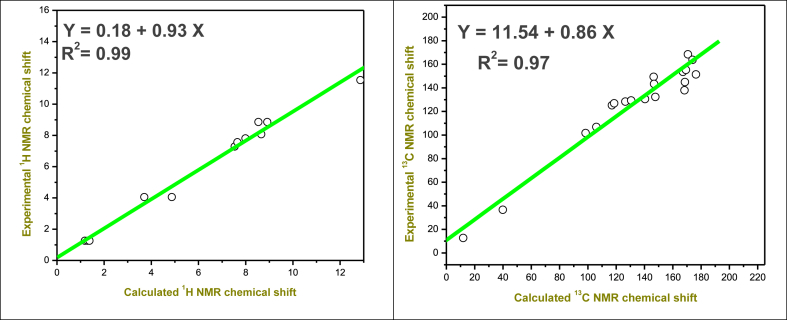
Correlation between the experimental and calculated chemical shifts for (a) ^1^H NMR and (b) ^1^³C NMR of **ATEHPQ.**

The compounds' ^1^H and ^13^C NMR spectra were recorded using TMS as an internal standard and DMSO as the solvent [38]. GIAO ^1^H and ^13^C chemical shift values (concerning TMS), were produced using the DFT (B3LYP) technique using the 6-311G++(d,p) basis sets, then these values were compared to the experimental chemical shift values. Table 2 presents the overall findings and experimental values from this computation. The theoretical and experimental ^1^H and ^13^C NMR spectra were visualized in Figs. S3 and S4. These chemical shift results were correlated graphically in Fig. 3. The Chemical shift value for OH is *δ* 11.54 ppm in the experimental section and *δ* 12.86 ppm in the computational section. The signal of NH_2_ is observed at *δ* 8.86 ppm whereas this signal is calculated at *δ* 8.54 and 8.92 ppm.

**Table 2:** Calculated and experimental ^1^H and^13^C NMR chemical shifts of **ATEHPQ** (**3**) on B3LYP/6–311++G(d,p) basis set.

^1^H NMR			^13^C NMR		
**Atoms**	Calculated	Experimental	Atoms	Calculated	Experimental
31-H	1.183	1.26	**28-C**	11.932	12.7
33-H	1.301	1.26	**27-C**	39.921	36.6
32-H	1.380	1.26	**20-C**	98.432	101.7
29-H	3.701	4.05	**11-C**	105.972	106.8
30-H	4.869	4.05	**4-C**	117.015	125.2
10-H	7.534	7.28	**2-C**	118.638	126.9
17-H	7.647	7.56	**6-C**	126.527	128.4
7-H	7.990	7.81	**5-C**	130.621	129.3
35-H	8.541	8.86	**1-C**	140.432	130.5
9-H	8.660	8.07	**15-C**	146.460	149.4
34-H	8.917	8.86	**26-C**	146.810	143.4
22-H	12.862	11.54	**3-C**	147.678	132.3
			**8-C**	167.192	153.6
			**23-C**	168.309	137.9
			**18-C**	174.030	163.8
			**12-C**	175.733	168.4

The C atoms of C=O_α–pyrone_ and C=O_quinoline_ group have signals of *δ* 174.03 and 175.73 ppm in the theoretical spectrum and *δ* 163.8 and 168.4 ppm in the experimental spectrum. The carbon atom of the ethyl group has theoretical chemical shifts at *δ* 11.93 ppm and 39.92 ppm and experimentally at *δ* 12.7 and 36.6 ppm.

### 3.5 Nonlinear optical analysis

Delocalization of the π-electron system in organic materials causes significant nonlinear responses [39]. DFT computational analysis was used to investigate the relationship between molecular structure and NLO properties [40]. NLO materials find widespread use in laser, telecommunications, optoelectronics, and photonics applications [41,42].

The calculated NLO parameters, including the dipole moment (μ), anisotropy of the polarizability (Δα), mean polarizability (α), and first-order hyperpolarizability (βtot) for the synthesized compounds, are summarized in Table 3. The results reveal that the dipole moment (μ) of these compounds is higher than that of the organic standard reference material, urea (μ = 2.3732 D). Additionally, the first-order hyperpolarizability (βtot) values are found to be 3–4 times greater than that of urea [43]. These synthesized compounds exhibit a strong NLO response.

**Table 3:** The dipole moment (μ), mean polarizability (α), anisotropy of the polarizability (Δα) and First-order hyperpolarizability (β) for compounds **1**–**3**.

Compound No.	μ_x_	μ_y_	μ_z_	μ_total_	<α> (au)	<α> (esu) X10^−23^	Δα (au)	Δα (esu) X10^−23^	β_total_ (au)	β_total_ (esu) X 10^−30^
**1**	6.8434	1.2905	−0.1188	6.97	123.35	1.83	14.96	0.222	150.84	1.303
**2**	2.0922	−2.8977	0.9745	3.70	139.75	2.07	84.28	1.25	156.57	1.353
**3**	−2.2737	−2.1794	0.9687	3.30	137.90	2.04	67.24	0.996	152.36	1.316

### 3.6 Drug likeness

ADME (absorption, distribution, metabolism, and excretion) investigation examines the physicochemical features of a possible medication candidate, which can be assessed utilizing online programs such as SWISSADME. The drug-likeness qualities of a molecule determine its potential for usage as a drug. Using Veber and Lipinski's criteria [[44], [45], [46]], these traits are utilized to predict the compounds' drug-like behavior. According to Lipinski, an orally active drug must match the following criteria: the octanol/water partition coefficient cannot be larger than 5, there must be no more than 10 hydrogen bond acceptors, and no more than 5 hydrogen bond donors [47]. Table 4 summarizes the physicochemical properties of the compounds under examination. Thus, those compounds have a molecular mass between 271 and 358 Da (MW), hydrogen bond donors (HBD) are in the range 1–3, and hydrogen bond acceptors (HBA) are found in the range 5–6 [47]. Further, the coefficient of partition octanol and water (LogP) is in the range of 1.03–1.64. In general, a negative log P value implies that the molecule has a high affinity for the aqueous phase, making it more hydrophilic. A positive log P value, on the other hand, implies that the molecule is more concentrated in the lipid phase and hence more lipophilic. Furthermore, research has shown that compounds with a log P value of less than five have a better chance of being absorbed. The compound's LogP partition coefficient was computed and found to be within the permissible range according to Lipinski's rule of five. This result reflects the degree of lipophilicity, which is critical for evaluating how a drug is distributed in the body after absorption [48].

**Table 4:** Lipinskiʼs and Veber's rules for drug-likeness of studied compounds **1**–**3**.

Compound	HBD	HBA	MW	Log P	TPSA	Rotatable bond	MR	Lipinski #violations	Veber violations
**1**	1	5	271.23	1.64	84.58	1	70.87	0	0
**2**	3	5	358.37	1.03	143.94	4	99.1	0	0
**3**	2	6	356.36	1.53	122.48	2	95.65	0	0

Additionally, Veber's rule states that a molecule with 10 or fewer rotatable bonds and a polar surface area (TPSA) of no more than 140 A°^2^ should have acceptable oral bioavailability. The topological polar surface area (TPSA) is a highly useful tool for predicting the transport properties of drugs [49]. The compounds' computed TPSA values vary from 84 to 134 A°^2^. This implies that, in theory, these compounds should not have problems with oral bioavailability.

### 3.7 Morphology characterizations

**Fig. 4:**
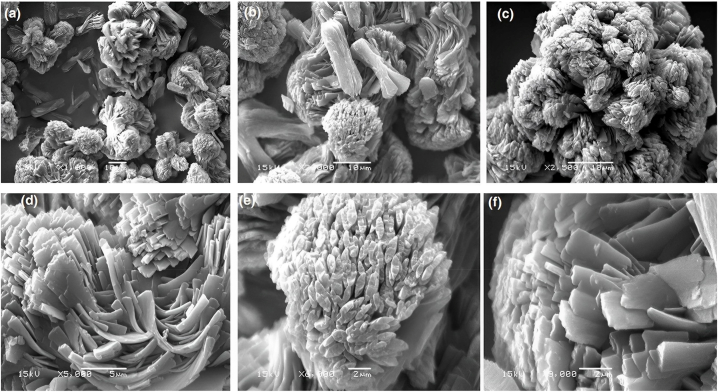
(a–f). SEM images showing the surface morphology of **ATEHPQ** at different magnifications.

Fig. 4(a–f) illustrates the surface morphology of **ATEHPQ** samples at various magnifications using scanning electron microscopy (SEM) prepared under optimized preparation conditions. The SEM images reveal a flower-shaped morphology with high agglomeration. These flower-like microspheres exhibited a solid core enveloped by loosely arranged thin nanosheets forming the outer layer. Nanoflowers are complex nanostructures composed of nanoscale petals or branches arranged in a flower-like pattern. This unique morphology contributes to a high surface area, which is advantageous for enhancing the interaction with light and improving charge carrier mobility. The intricate petal-like structures can trap light more effectively, leading to higher absorption and, consequently, better photovoltaic performance. In the context of solar cells, such nanostructures can improve light-harvesting efficiency and facilitate faster electron transport, reducing recombination losses [50].

Compared to other organic structures used in solar cells, **ATEHPQ** nanoflowers exhibit distinct advantages. Traditional organic solar cell materials often suffer from limited light absorption and suboptimal charge carrier mobility. The flower-like morphology of **ATEHPQ** enhances light absorption due to its high surface area and improves the pathways for charge carriers, thereby increasing the overall efficiency of the solar cells. Additionally, the complex nanostructures of **ATEHPQ** provide better mechanical stability and can be more easily integrated into flexible solar cell designs, making **ATEHPQ** a promising candidate for next-generation organic solar cells. In comparison, acridine-based metal-free organic dyes for dye-sensitized solar cells, as studied by Öztürk et al. [51], may not achieve the same level of mechanical stability and flexibility as **ATEHPQ**. Similarly, the Au/Carmine/n-Si/Ag diode structure investigated by Bodur et al. [52] offers unique photovoltaic and photodiode properties, but **ATEHPQ**'s nanoflower morphology potentially provides enhanced light absorption and charge carrier mobility, leading to improved solar cell performance. Similar improvements in solar cell efficiency have been observed in studies such as those by Z. Samavati et al., where alterations in nanostructure morphology significantly impacted the performance of solar cells [50].

The enhanced performance of **ATEHPQ**-based solar cells can be attributed to the unique nanoflower morphology observed in the SEM images [[53], [54], [55]]. The high surface area provided by the nanoflowers allows for greater light absorption and interaction, which is crucial for photovoltaic applications. Additionally, the complex structure aids in efficient charge separation and transport, minimizing recombination losses. When compared to other organic materials, **ATEHPQ** stands out due to its superior light-harvesting capabilities and improved charge carrier mobility, which are essential for high-efficiency solar cells. The findings from this study highlight the potential of **ATEHPQ** nanoflowers in advancing organic solar cell technology and opening up new avenues for the development of highly efficient, flexible, and stable photovoltaic devices [[52], [53], [54], [55], [56], [57]].

**Fig. 5:**
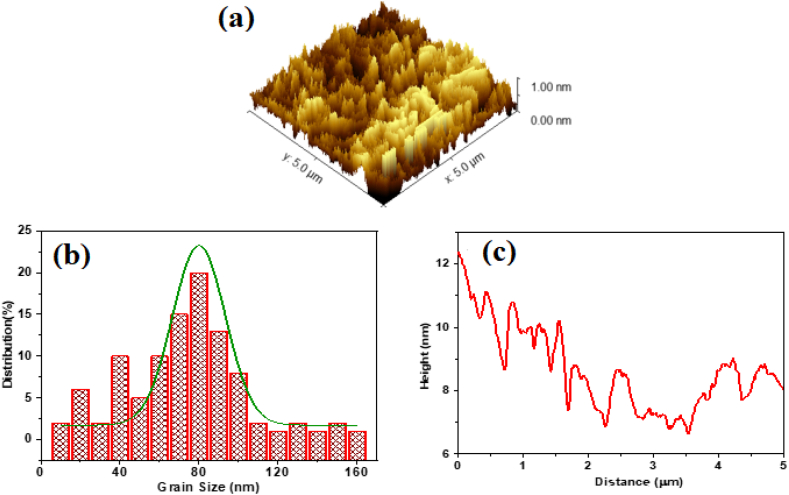
(a) 3D -SEM image, (b) Histogram of grain size distribution, and (c) Roughness tracer investigation of **ATEHPQ** thin films.

In the analysis of **ATEHPQ** thin films, the application of advanced imaging techniques provides critical insights into the material's structural properties. Utilizing 3D Scanning Electron Microscopy (SEM), shown in Fig. 5(a), detailed images were captured, revealing the three-dimensional arrangement of grains within the film. The histogram of grain size distribution, shown in Fig. 5(b), obtained through Gwyddion software, shows an average grain size of approximately 80.6 nm, indicating a relatively uniform distribution of grain sizes. Furthermore, roughness tracer analysis, conducted with ImageJ, reveals that the roughness of the thin films ranges from 6 to 10 nm as indicated in Fig. 5(c). These measurements are crucial for understanding the film's surface quality and its potential impact on device performance.

When comparing these properties to other organic structures used in solar cells, **ATEHPQ** thin films exhibit a fine grain structure and moderate roughness. For instance, organic photovoltaic materials like P3HT [56] often show larger grain sizes and varying roughness, which can influence charge transport and overall efficiency. The relatively small grain size and controlled roughness of **ATEHPQ** thin films suggest they could offer advantages in creating more uniform films with potentially better performance characteristics in solar cell applications. Precise control over grain size and surface roughness is essential for optimizing the efficiency of organic solar cells and ensuring consistent performance across large-scale devices [[51], [52], [53], [54], [55]].

**Fig. 6:**
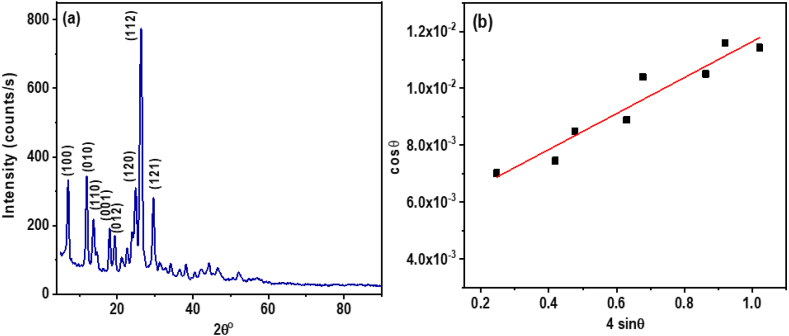
(a) X-ray diffraction (XRD) pattern, and (b) Graph of cos θ plotted against 4 sin θ for **ATEHPQ** thin films.

Fig. 6(a) showcases the polycrystalline nature of **ATEHPQ**, highlighting its well-defined crystalline structure. The X-ray diffraction (XRD) data obtained for **ATEHPQ** was processed using the CRYSFIRE program, which confirmed that the material adopts a monoclinic crystal system. This detailed analysis was further refined using the Check cell program, which determined the corresponding Miller indices (h k l) for each diffraction peak as (100), (010), (110), (001), (012), (120), (112), and (121). These indices provide valuable insights into the orientation and arrangement of crystal planes. Such structural information is crucial for applications in solar cells, where the polycrystalline nature and specific monoclinic crystal system of **ATEHPQ** can enhance charge carrier mobility and reduce recombination losses, leading to higher efficiency. Compared to other organic structures used in solar cells, which might have different crystal systems or amorphous characteristics, **ATEHPQ**'s well-defined grain boundaries and crystal orientation offer distinct advantages in performance and stability. The detailed crystallographic analysis presented in Fig. 6(a) underscores **ATEHPQ**'s potential as a promising material for solar cell applications, providing a solid foundation for improved efficiency and durability in organic photovoltaic materials [57].

By utilizing the well-established Williamson-Hall plot as stated in the literature [58] for all identified planes, as shown in Fig. 6(b), we determined the average crystal size and lattice strain of the material. The Williamson-Hall analysis provided insights into the strain distribution across various crystallization planes, revealing a nearly uniform strain distribution under typical deformation. This uniformity indicates isotropy within the crystal structure, a desirable attribute for many applications. The calculated average values for crystallite size and strain were 26.08 nm and 6.3 × 10^−3^, respectively. These measurements are crucial for understanding the material's mechanical properties and stability, which directly influence its performance in practical applications.

When comparing this material to other organic structures used in solar cells, the uniform strain distribution and small crystallite size of 26.08 nm offer significant advantages. In solar cells, such characteristics can enhance charge carrier mobility by providing consistent pathways for electron flow and minimizing recombination losses. Other organic materials might not exhibit the same level of isotropy and could have larger or more irregular crystallites, potentially leading to inefficiencies. The precise control over crystal size and strain in this material underscores its potential for high-performance solar cell applications. By optimizing these structural parameters, the material can improve light absorption, charge transport, and overall device efficiency, making it a strong candidate for next-generation solar technologies.

### 3.8 Thermogravimetric characterization

**Fig. 7:**
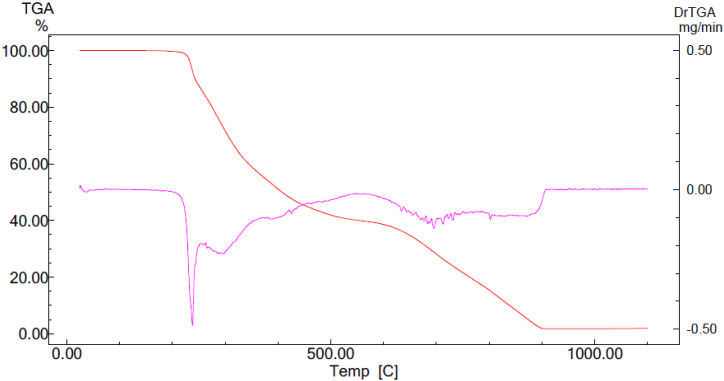
TGA diagram of the synthesized **ATEHPQ**.

The thermogram depicted in Fig. 7 illustrates the heating profile of the **ATEHPQ** sample within the temperature range of 300–1100 K at a rate of 10 K/min. In the initial region of the thermogravimetric analysis (TGA) curve, spanning approximately from 323 to 340 K, a minor weight loss is observed as the temperature increases. This initial weight loss is attributed to the evaporation of volatile gases that were adsorbed by the material from the surrounding atmosphere during processing. Such behavior is typical for materials that have undergone exposure to ambient conditions, resulting in the adsorption of moisture and other volatile components. This initial phase indicates that the sample sheds these loosely bound volatiles before any significant thermal events occur. As the temperature surpasses 513 K (240 °C), a more substantial weight loss is evident, indicating a major thermal event. This significant weight loss corresponds to the melting temperature and subsequent decomposition of the **ATEHPQ** structure. The sharp decline in weight at this stage signifies that the material undergoes a phase transition, followed by thermal decomposition. This behavior confirms the thermal stability of the **ATEHPQ** up to this critical temperature. The material remains stable with minimal weight loss up to the threshold where it begins to decompose. This stability is crucial for applications where the material might be subjected to elevated temperatures, such as in solar cells, where thermal stability ensures longevity and consistent performance [55]. The distinct phases observed in the TGA curve highlight the robustness of **ATEHPQ** in maintaining its integrity under thermal stress, validating its potential for high-temperature applications.

### 3.9 Photoluminescence characterization

**Fig. 8:**
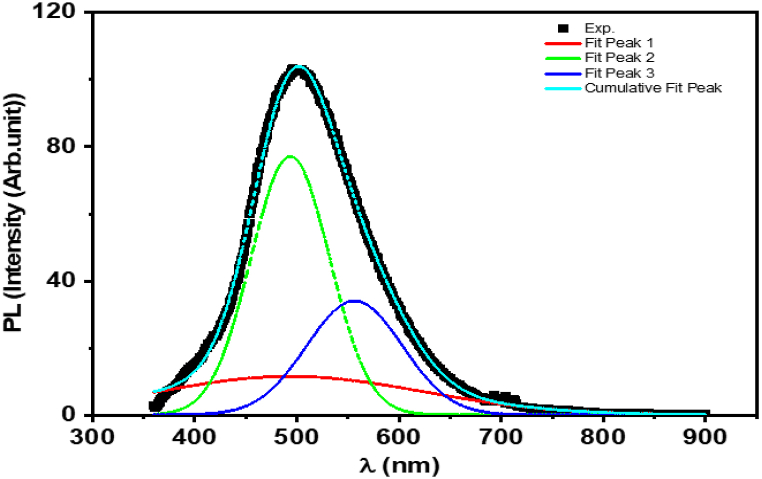
Spectral variation of photoluminescence in **ATEHPQ** thin films.

The photoluminescence (PL) spectrum of **ATEHPQ**, as depicted in Fig. 8, reveals a complex emission profile characterized by a main peak centered around 502 nm. This main peak is further dissected into well-defined sub-bands, which indicate the presence of molecular vibrations affecting the emission process. Each of these sub-bands can be accurately described using Lorentzian functions, which are mathematical representations ideal for depicting natural linewidths broadened by homogenous processes. These sub-bands signify slightly different energy level transitions that occur during the relaxation of excited states back to the ground state. The observation of these sub-bands within the PL spectrum highlights the intricate interplay between electronic states and vibrational modes in the **ATEHPQ** molecule, suggesting that the emission process is not a single, straightforward transition but rather a combination of multiple, closely related transitions.

The detailed analysis of these sub-bands provides valuable insights into the photophysical properties of **ATEHPQ**. The presence of such well-defined sub-bands implies a high degree of structural order and uniformity within the material, which is crucial for optoelectronic applications such as solar cells. The distinct energy level transitions observed suggest that **ATEHPQ** has a robust mechanism for absorbing and re-emitting light, which can be harnessed to improve the efficiency of solar cells. By optimizing these transitions, it is possible to enhance the material's light-harvesting capabilities and charge carrier dynamics, leading to better performance. Moreover, the ability to precisely describe these sub-bands using Lorentzian functions allows for accurate modeling and prediction of the material's behavior under various conditions, further aiding in the design of high-efficiency solar cells and other optoelectronic devices [59].

The photoluminescence (PL) spectra comparison between our results for **ATEHPQ** and the substituted 2-(1H-benzimidazol-2-yl)quinoline derivatives reported by Sahin et al. [60] reveals distinct differences in their emission characteristics. The substituted 2-(1H-benzimidazol-2-yl)quinoline derivatives exhibit broad emission bands with peaks centered at 634, 645, and 636 nm, indicating longer wavelength emissions in the red region. In contrast, **ATEHPQ** displays a complex emission profile with a main peak centered around 502 nm, situated in the green region, and further dissected into well-defined sub-bands suggesting molecular vibrations. The differences in emission peaks and profiles can be attributed to the distinct molecular structures and substituents of the compounds. The bathochromic shift in one of the substituted 2-(1H-benzimidazol-2-yl) quinoline derivatives is due to the electron-donating properties of the methyl groups, which influence the electronic environment and thus the emission wavelength. Conversely, **ATEHPQ**'s complex emission with sub-bands indicates a more intricate interaction of molecular vibrations with the electronic states, which is not as prominently observed in the simpler broad emissions of the substituted 2-(1H-benzimidazol-2-yl) quinoline derivatives. These structural variations and substituents lead to the observed disparities in their PL spectra [60].

### 3.10 Absorption characterizations

**Fig. 9:**
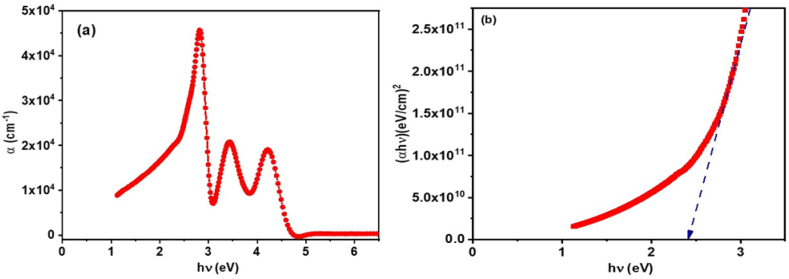
(a) Plot of absorbance *versus.* hν, and (b) Graph of (αhν)^2^ plotted against photon energy (hν) for **ATEHPQ** thin films.

The optical properties of **ATEHPQ** thin films are elucidated through their absorbance spectrum and the analysis of their energy band gap. The plot of absorbance versus energy, as depicted in Fig. 9(a), reveals distinct peaks at 2.8 eV, 3.41 eV in the visible region, and 4.21 eV in the ultraviolet (UV) region. These peaks indicate the presence of strong absorption bands corresponding to electronic transitions within the material. The absorption peaks in the visible range suggest that **ATEHPQ** thin films are highly effective in absorbing visible light, making them suitable for applications in optoelectronic devices such as photovoltaic cells and light-emitting diodes (LEDs). The peak at 4.21 eV in the UV region further implies that the material can also be employed in UV-sensitive applications, including UV photodetectors and protective coatings.

Using Tauc's model relations, one can determine both the values of the indirect optical band gap and the type of electronic transition, specifically from the highest occupied molecular orbital (HOMO) to the lowest unoccupied molecular orbital (LUMO). This approach provides valuable insights into the material's electronic properties and is expressed as follows [47]:

(1) $${\left(\alpha h\nu \right)}^{2}=B\left(h\nu -E_{g}\right)$$

In the context where *h* represents Planck's constant, *B* is the proportionality constant, and *E*_g_ denotes the energy bandgap.

The plot of (*αhν*)^2^ versus energy (hν) for **ATEHPQ** thin films, shown in Fig. 9(b), provides insight into the electronic transition and the material's band gap. The linear region of the plot indicates a direct transition, with an energy gap of approximately 2.43 eV. This direct band gap is a crucial parameter, as it determines the efficiency of electron-hole pair generation when the material absorbs photons. A direct band gap of 2.43 eV suggests that **ATEHPQ** thin films can efficiently convert absorbed light into electrical energy, making them highly suitable for photovoltaic applications. Additionally, the band gap in this range allows for efficient emission of light, which is beneficial for applications in LEDs and other light-emitting devices. The direct nature of the energy gap in **ATEHPQ** thin films enhances their performance in photovoltaic devices by allowing efficient absorption of photons and direct electron transitions from the valence to the conduction band. This reduces recombination losses and improves charge carrier generation, leading to better light absorption and conversion efficiency in solar cells. As a result, **ATEHPQ** thin films exhibit superior photovoltaic performance compared to materials with indirect band gaps. Moreover, the energy gap of 2.43 eV is significant for **ATEHPQ** thin films because it falls within the ideal range for efficient light absorption in the visible spectrum, which is critical for photovoltaic applications. A well-suited bandgap allows the material to effectively convert sunlight into electrical energy. In this case, the energy gap enables a balance between sufficient light absorption and minimizing energy losses, contributing to higher open-circuit voltage and improved overall efficiency in thin-film solar cells.

In comparison to the study by El-Ghamaz et al. [61], which reported absorbance bands for azodye derivatives, the **ATEHPQ** thin films exhibit different optical properties. El-Ghamaz et al. identified absorption bands at 3.0–3.5 eV, attributed to n–π∗ transitions of azodye derivatives' N=N functional groups, and bands at 5.0–5.2 eV, assigned to π–π∗ transitions between orbitals localized in the phenyl ring. Furthermore, the energy gap for the azodye derivatives was reported to be 3.27 eV. In contrast, **ATEHPQ** thin films show distinct absorption peaks and a lower energy gap of 2.43 eV, suggesting different electronic structures and transitions. These differences highlight the unique optical properties of **ATEHPQ** thin films, making them more versatile for specific optoelectronic applications.

### 3.11 Dispersion characteristics

**Fig. 10:**
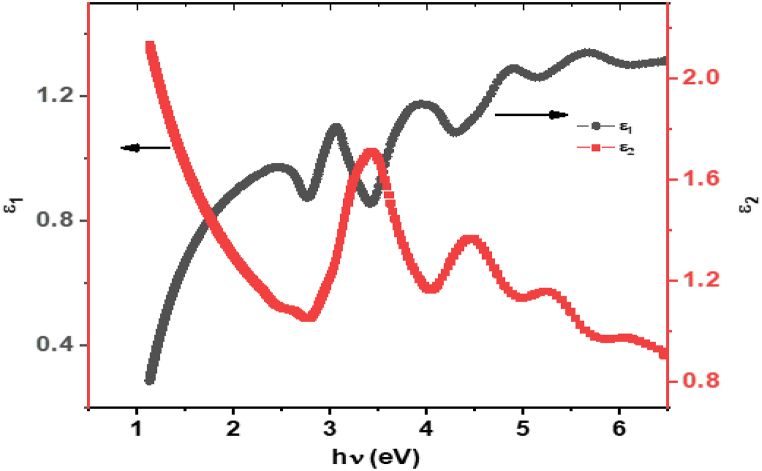
The dependence of *ε*_1_ and *ε*_2_ on photon energy for **ATEHPQ** thin films.

The photon energy dependence of the dielectric function, including both the real part (*ε*₁) and the imaginary part (*ε*₂), is illustrated in Fig. 10. This figure reveals how the dielectric properties of **ATEHPQ** thin films vary with photon energy, highlighting the complex relationship between energy absorption and the material's response. The real part of the dielectric function, *ε*₁, typically represents the material's ability to store electrical energy within an electric field, while the imaginary part, *ε*₂, corresponds to the material's absorption characteristics. Analyzing these components provides insight into how **ATEHPQ** thin films interact with electromagnetic waves across different energy ranges. In Fig. 10, the real part of the dielectric function *ε*₁ shows variations with photon energy, indicating changes in the material's optical conductivity. Peaks in *ε*₁ generally correspond to resonance conditions where the material effectively interacts with the incident light, leading to enhanced electrical polarization. These resonances can be associated with specific electronic transitions within the **ATEHPQ** thin films. The behavior of *ε*₁ as a function of photon energy reveals how well the material can polarize in response to an external electric field, which is crucial for understanding its potential applications in optical and electronic devices.

Conversely, the imaginary part *ε*₂ depicts how efficiently the **ATEHPQ** thin films absorb photons at various energies. Peaks in *ε*₂ signify wavelengths where the material has strong absorption, leading to significant energy dissipation through non-radiative processes. This absorption spectrum is vital for applications where high absorption efficiency is required, such as in photodetectors or light-harvesting systems. The interplay between *ε*₁ and *ε*₂ across photon energies highlights the comprehensive optical properties of **ATEHPQ** thin films, demonstrating their suitability for various optoelectronic applications where both storage and absorption of optical energy are critical factors.

**Fig. 11:**
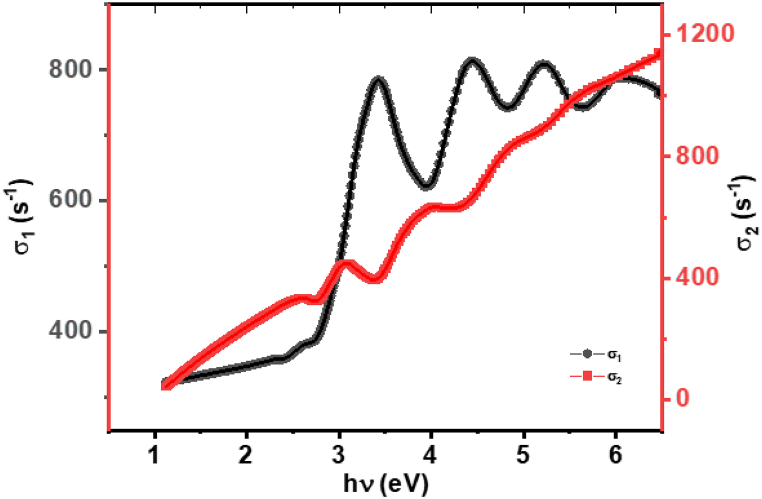
Plot of σ_1_ and σ_2_*versus* hν of **ATEHPQ** thin films.

Fig. 11 illustrates the relationship between the optical conductivity components, σ₁ (real part) and σ₂ (imaginary part), and photon energy (*E*) for **ATEHPQ** thin films. The plot reveals how these components vary with energy and are closely related to the dielectric constant of the material. The real part of optical conductivity, σ₁, is indicative of the material's ability to conduct electric current under the influence of an alternating electric field. It shows how the material's conductivity changes with energy, reflecting its response to electromagnetic radiation. In contrast, the imaginary part, σ₂, provides insight into the energy absorbed by the material, which is associated with electronic transitions and the material's ability to dissipate energy.

The variations in σ₁ and σ₂ with photon energy are directly connected to the dielectric constant of **ATEHPQ** thin films. Specifically, the dielectric constant is derived from the relationship between these conductivity components and the material's response to electric fields. Peaks in σ₂, which correspond to significant absorption features, indicate regions where the material efficiently absorbs energy, thus influencing the overall dielectric behavior. Conversely, changes in σ₁ reflect how the material's conductive properties are altered across different energies. Understanding these relationships helps in evaluating the material's suitability for various optoelectronic applications, as it provides a comprehensive view of how the material interacts with electromagnetic fields and its overall performance in practical devices.

The single oscillator model is an effective approach for simplifying the complex interaction between light and matter by representing it with a single resonant frequency. This model facilitates the estimation of key optical parameters, such as the oscillator strength (E₀) and dispersion energy (Eᴅ), and the expression as follows:

(2) $${\left(n^{2}-1\right)}^{-1}=\frac{E_{o}^{2}-E^{2}}{E_{o}E_{d}}$$

**Fig. 12:**
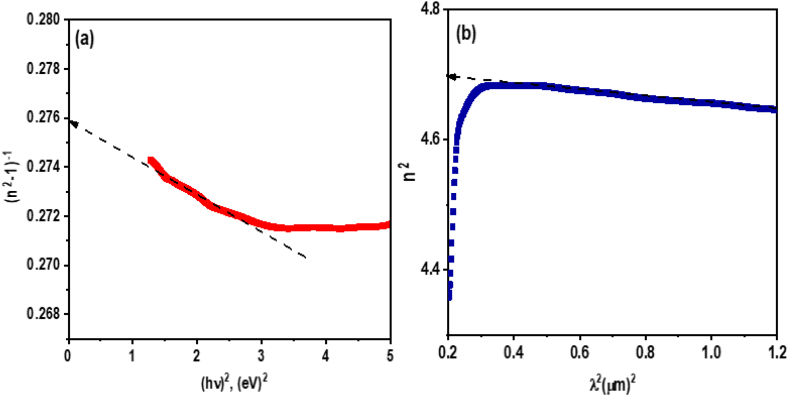
Plot of (a) (n^2^ -1)^−1^*versus* (hν)^2^, and (b) n^2^*versus* λ^2^ of **ATEHPQ** thin films.

By approximating the material with a single effective resonance frequency, the model allows for straightforward calculation of these parameters, offering valuable insights into the material's optical behavior. For **ATEHPQ** thin films, the application of this model, as illustrated in Fig. 12(a), has provided an oscillator energy of 3.12 eV and a dispersion energy of 14.21 eV. These parameters are crucial for understanding how the material interacts with light and for predicting its performance in practical applications, such as in solar energy technologies.

The Wemple-DiDomenico relation extends the utility of the single oscillator model by correlating the optical constants derived from it with the material's electronic structure and microstructure. This relation helps bridge the gap between empirical optical data and theoretical understanding, providing a framework to interpret how structural and electronic characteristics influence optical properties. The estimated oscillator energy of 3.12 eV and dispersion energy of 14.21 eV suggest that **ATEHPQ** thin films have favorable optical properties for solar energy applications. Specifically, these values indicate that the material has a significant absorption range and effective dispersion, making it suitable for capturing and converting solar energy efficiently. This aligns with the needs of photovoltaic technologies, where materials with appropriate optical and electronic properties are crucial for enhancing energy conversion efficiency.

In our investigation, we applied fundamental principles of electromagnetic theory to examine the optical properties of **ATEHPQ** thin films. We specifically focused on the relationship between the refractive index squared (n^2^) and the square of the wavelength (λ^2^), as illustrated in Fig. 12(b) and expressed in the following:

(3) $$n^{2}=\varepsilon_{\infty }-\frac{e^{2}}{4\pi^{2}\varepsilon_{o}c^{2}}\lambda^{2}$$

This approach allowed us to derive key optical parameters from the refractive index data, providing insights into the material's dispersion characteristics. From this analysis, we determined that the high-frequency dielectric constant for **ATEHPQ** thin films is 4.71. This value reflects the material's capability to polarize in response to an electric field, which is crucial for understanding its optical performance and potential applications in optoelectronic devices.

The molecular architecture of the **ATEHPQ** compound, characterized by a non-planar structure and a benzene ring displaced from the molecular plane, is anticipated to have a profound influence on the distribution of electrons within the molecule. This structural deviation, coupled with the molecule's extensive conjugation and the electron-donating properties of the two amino groups, is expected to enhance the molecule's polarizability. This heightened polarizability is crucial for optimizing nonlinear optical (NLO) properties, facilitating efficient charge transport through the creation of low-energy molecular orbitals. Given the remarkable characteristics of organic dyes, they have garnered significant attention for their potential applications in a wide range of photonic and optoelectronic technologies, including solar cells, lasers, displays, telecommunications, and optical data storage.

The interaction between the **ATEHPQ** film and incident light results in polarization, which can be linearly proportional to the electric field, leading to linear optical susceptibility (χ^(1)^). However, under specific conditions, higher-order nonlinearities emerge, represented by third-order nonlinear optical susceptibility (χ ^(3)^). The linear optical susceptibility can be quantitatively determined using established models and equations proposed as follows [62]:

(4) $$\chi^{\left(1\right)}=\frac{n^{2}-1}{4\pi }$$

Moreover, the third-order nonlinear optical susceptibility, χ ^(3)^ can be calculated as

(5) $$\chi^{\left(3\right)}=A{\left(\frac{n^{2}-1}{4\pi }\right)}^{4}$$

**Fig. 13:**
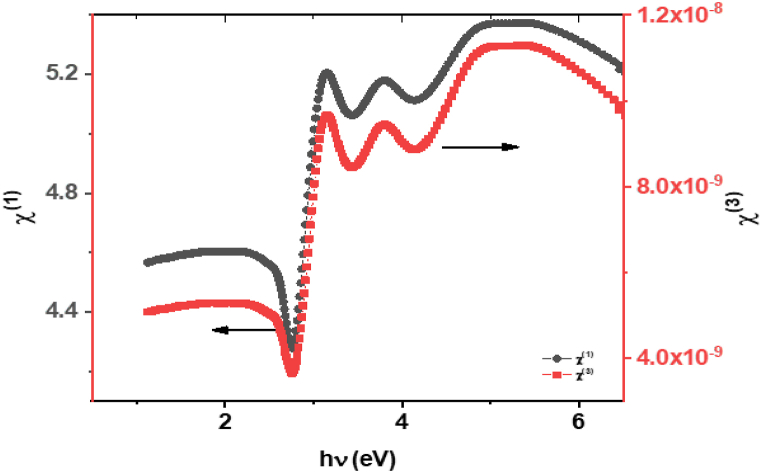
Plot illustrating the variation of χ^(1)^ and χ^(3)^ as a function of hv for ATEHPQ thin films.

Fig. 13 presents a graphical representation of the linear (χ^(1)^) and nonlinear (χ^(3)^) optical susceptibilities of **ATEHPQ** thin films as a function of the applied electric field (E). This visual depiction provides valuable insights into the material's optical response under varying electrical conditions. The nonlinearity observed in χ^(3)^ is attributed to the material's ability to generate new frequencies of light in response to intense incident radiation. This phenomenon arises from the interaction between the strong electric field of the light and the electronic structure of the material, inducing a nonlinear polarization response.

The values of the third-order susceptibility (χ^(3)^) and the nonlinear refractive index (n_2_) for **ATEHPQ** thin films are consistent with published results in the literature. Studies have shown that the relationship between χ^(3)^ and n_2_ is well-documented, with both parameters being crucial for understanding the nonlinear optical properties of materials. For instance, research published in the Journal of the Optical Society of America B discusses the correlation between χ^(3)^ and *n*_2_, highlighting that the experimentally determined values align with theoretical predictions1. Additionally, materials for third-order nonlinear optics, as reviewed in MRS Bulletin, confirm that the observed χ^(3)^ values for various materials, including **ATEHPQ** thin films, agree with the nonlinear refractive index measurements2. This consistency across different studies reinforces the reliability of the experimental methods and the robustness of **ATEHPQ** thin films in nonlinear optical applications.

A comparative analysis of the data presented in Fig. 13 with existing literature [62] reveals a consistent pattern, thereby validating the accuracy and reliability of the obtained results. This corroboration strengthens the credibility of the findings and underscores the material's potential for applications in nonlinear optics.

**Fig. 14:**
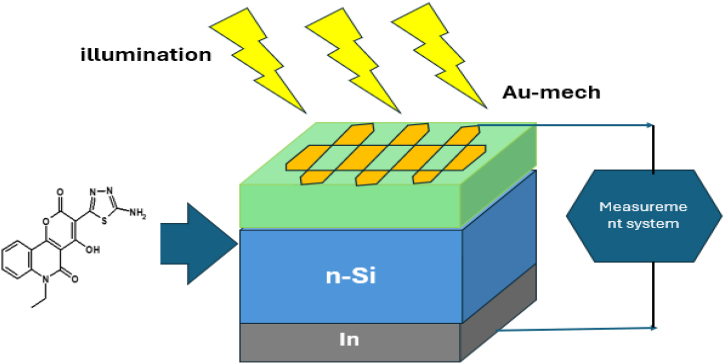
A schematic representation of the heterojunction diode structure based on **ATEHPQ.**

Fig. 14 provides a schematic representation of the Au/**ATEHPQ**/n-Si heterojunction structure. This diagram illustrates the layered configuration, featuring a gold (Au) contact, an **ATEHPQ** organic semiconductor layer, and an n-type silicon (n-Si) substrate. The gold layer serves as the electrode, facilitating electrical contact with the **ATEHPQ** film. The **ATEHPQ** layer acts as the active organic material, positioned between the gold contact and the n-type silicon substrate. The n-Si layer forms the base of the heterojunction, providing the necessary electronic properties for the device's functionality. This schematic highlights the interfaces and material interactions within the heterojunction, crucial for understanding its performance in electronic and optoelectronic applications. The arrangement underscores the integration of organic and inorganic materials to achieve desired electronic characteristics, leveraging the strengths of both components in the heterojunction design [61].

**Fig. 15:**
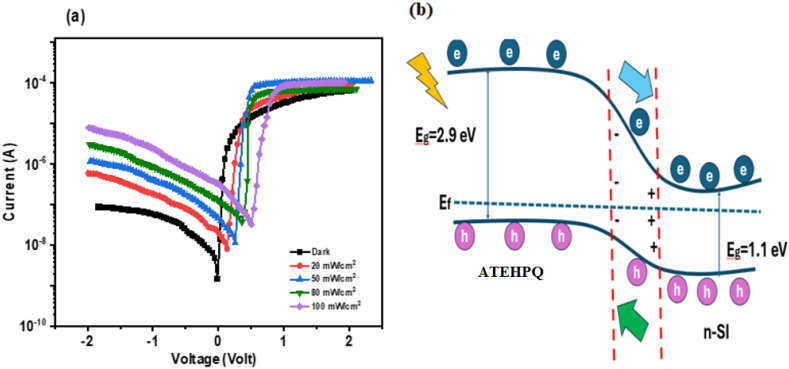
(a) Plot of current-voltage (I-V characteristics), and (b) Schematic band diagram of **ATEHPQ**-based heterojunction diode structure.

Fig. 15(a) shows the current-voltage (I-V) characteristics of the **ATEHPQ**-based heterojunction under different illumination intensities: 20, 50, 80, and 100 mW/cm^2^. The plot reveals that as the illumination intensity increases, the reverse bias current rises more sharply compared to the forward bias current. This trend indicates that the device is highly sensitive to changes in light intensity when operated in reverse bias. The increased reverse bias current suggests enhanced photogenerated charge carrier collection efficiency, which is crucial for photovoltaic applications. The higher reverse bias current under greater illumination intensities implies that the heterojunction effectively converts more light into electrical energy. This increased sensitivity is advantageous for applications in light detection and solar cells, where the goal is to maximize the conversion of light into useable electrical power [60,61].

Fig. 15(b) provides a schematic diagram of the **ATEHPQ**-based heterojunction structure, illustrating its layered configuration. The diagram shows the **ATEHPQ** organic semiconductor layer sandwiched between metal and semiconductor layers. In this structure, the **ATEHPQ** layer plays a critical role as the active organic material that interacts with incident light. The adjacent metal (e.g., Au) and semiconductor layers (e.g., n-Si) form the heterojunction, facilitating efficient charge transfer. This arrangement is crucial for the photovoltaic characteristics of the device, as it determines how effectively the heterojunction absorbs light and separates charge carriers. The schematic helps visualize how the different layers work together to enhance light absorption and electrical conductivity, contributing to the overall efficiency of solar cells. Understanding the layout and function of each layer is essential for optimizing the heterojunction for photovoltaic applications, ensuring effective light conversion and energy production.

**Fig. 16:**
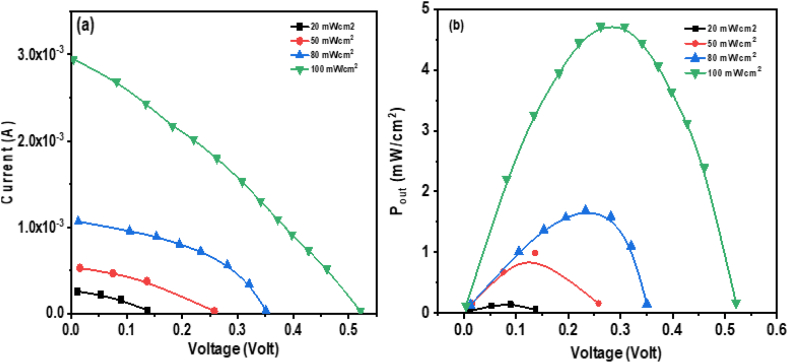
(a) Plot of current-voltage (I–V) characteristics, and (b) Plot of P_out_*versus* voltage of **ATEHPQ** -based heterojunction diode structure under various illuminations.

Fig. 16(a) depicts the current-voltage (I-V) characteristics of the **ATEHPQ**-based heterojunction structure under varying illumination intensities of 20, 50, 80, and 100 mW/cm^2^. The plot reveals distinct trends in both short-circuit current and open-circuit voltage as illumination increases. Specifically, the short circuit current, which represents the maximum current flowing through the device when the output terminals are shorted, increases with higher light intensities. This behavior indicates that the device is effectively generating more charge carriers as the light intensity rises [60]. Conversely, the open circuit voltage, the maximum voltage across the device when no current flows, also shows an increase with illumination but to a lesser extent. This increase in both parameters reflects improved device performance with higher light levels, demonstrating that the **ATEHPQ**-based heterojunction can efficiently convert light into electrical energy and maintain enhanced voltage and current output under increased illumination conditions. In the context of photovoltaic devices, Voc (open-circuit voltage) represents the maximum voltage a device can produce when no current is flowing, while Isc (short-circuit current) refers to the maximum current generated when the voltage is zero. FF (fill factor) is a measure of the quality of the solar cell, defined as the ratio of the maximum power output to the product of Voc and Isc. These parameters are crucial because they directly influence the overall efficiency (η) of the solar cell, with higher values of Voc, Isc, and FF leading to better performance and higher energy conversion efficiency.

Fig. 16(b) presents a plot of output power versus voltage for the **ATEHPQ**-based heterojunction structure. The graph highlights the relationship between the device's output power and the applied voltage, showing a distinct maximum power value. This peak in the output power curve indicates the optimal operating point of the heterojunction structure, where the balance between voltage and current is maximized to deliver the highest power output [61]. The presence of this maximum power point suggests that the device can be optimized for efficient energy conversion by operating near this peak. Analyzing this plot is crucial for understanding the efficiency of the **ATEHPQ**-based heterojunction in practical applications, as it helps in determining the optimal operating conditions for maximizing power output. The ability to achieve a significant output power indicates the potential of the heterojunction structure for use in energy-harvesting applications where high efficiency is required.

**Fig. 17:**
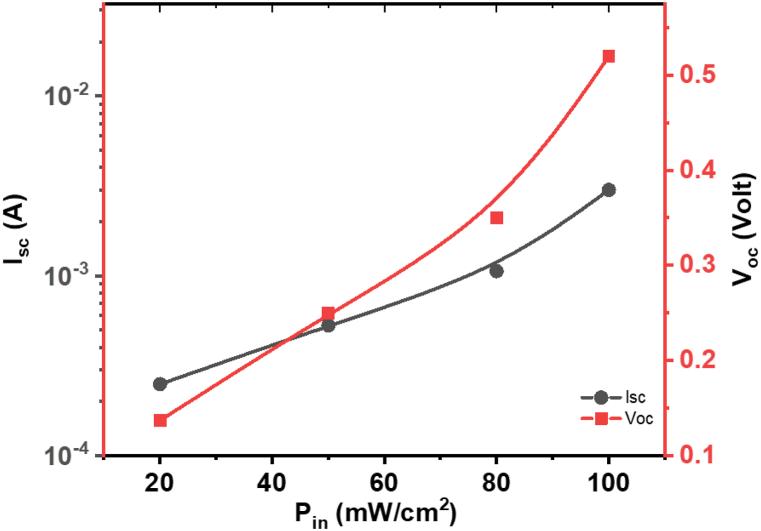
Plot of I_sc_*versus* P_in_ (left side), and V_oc_*versus* P_in_ (right side) of **ATEHPQ**-based heterojunction diode structure.

Fig. 17 illustrates the performance characteristics of the **ATEHPQ**-based heterojunction structure under different illumination intensities. Fig. 17 (left side) shows the plot of short circuit current (I_sc_) versus incident power (P_in_), while the right side of Fig. 17 displays the plot of open circuit voltage (V_oc_) versus P_in_. Both plots reveal a positive correlation between illumination intensity and the respective electrical parameters. Specifically, I_sc_ increases with higher P_in_, reflecting the enhanced generation of charge carriers as the light intensity grows. Similarly, V_oc_ also rises with increased P_in_, indicating that the device's ability to maintain a higher voltage under illumination improves with higher light levels. These observations suggest that the **ATEHPQ**-based heterojunction is effective at converting light into electrical energy, with both current and voltage benefiting from increased illumination [60,61].

The increase in both I_sc_ and V_oc_ with higher illumination intensities demonstrates the potential of the **ATEHPQ**-based heterojunction for practical applications in photovoltaic devices [61]. The short-circuit current (Isc) is crucial in determining a photovoltaic cell's performance, as it represents the maximum current generated under illumination when no external load is applied. A higher Isc indicates greater light absorption and more efficient charge carrier generation, directly influencing the cell's power output. Factors affecting Isc include the intensity and wavelength of incident light, the material's absorption properties, the surface area of the cell, and the quality of the junction, which can be impacted by recombination losses and defects. Enhanced I_sc_ indicates that the device can generate more electrical current under higher light conditions, which is crucial for efficient solar energy conversion. The rise in V_oc_ suggests improved voltage output, contributing to better overall power generation. These characteristics are particularly valuable for applications in solar cells, where maximizing both current and voltage is essential for achieving high energy conversion efficiency. The ability to sustain high I_sc_ and V_oc_ under varying light conditions highlights the suitability of this heterojunction structure for solar energy harvesting and other optoelectronic applications, where reliable and efficient performance under different illumination levels is required.

**Fig. 18:**
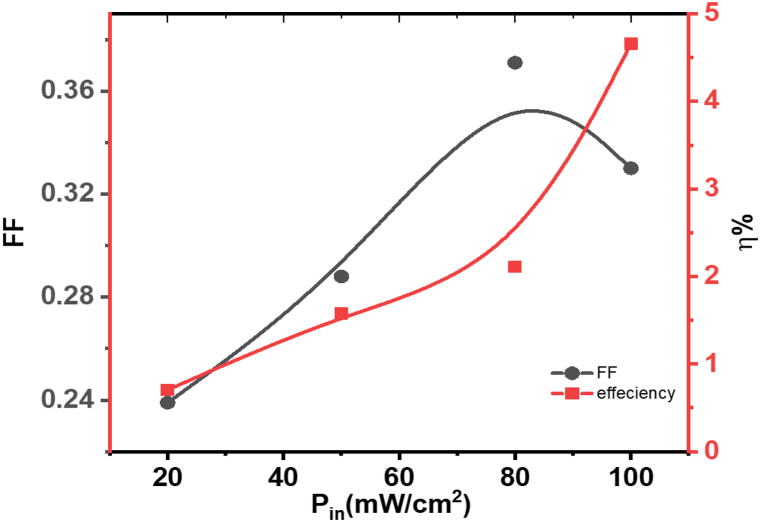
The relationship between the fill factor (FF) and input power (P_in_) on the left, and the relationship between efficiency (η) and input power (P_in_) on the right for the ATEHPQ-based heterojunction diode structure.

Fig. 18 provides insights into the performance of the **ATEHPQ**-based heterojunction structure by plotting the fill factor (FF) versus incident power P_in_ in Fig. 18 (left side) and conversion efficiency versus P_in_ in the right side of Fig. 18. The fill factor, a measure of the quality of the solar cell, is observed to vary with different levels of illumination. As shown, FF generally increases with higher illumination intensities, which suggests improved device performance under increased light conditions. This trend indicates that the heterojunction structure maintains a higher proportion of its maximum power output relative to the theoretical maximum, demonstrating its efficiency in converting light into electrical power. Fig. 18 depicts how conversion efficiency, the ratio of electrical power output to incident light power, varies with P_in_. The plot shows an increase in conversion efficiency with higher illumination levels, reflecting the device's ability to effectively convert light into electrical energy as the light intensity increases. The open-circuit voltage (V_oc_) is directly related to the overall efficiency of a photovoltaic cell, as it contributes to the maximum power output of the device. A higher V_oc_ typically leads to improved energy conversion efficiency. Factors affecting V_oc_ include the material's band gap (a larger band gap generally results in a higher V_oc_), the quality of the material (defects or impurities can reduce V_oc_ by causing recombination losses), and the temperature (higher temperatures can decrease V_oc_ due to increased carrier recombination).

Comparing these results with other organic-based heterojunctions [[60], [61], [62]], the **ATEHPQ**-based device demonstrates competitive performance. In general, higher fill factors and conversion efficiencies indicate better overall efficiency in light-to-electricity conversion. When compared to similar devices, such as those utilizing other organic semiconductors, the **ATEHPQ**-based heterojunction holds its ground in efficiency metrics, thanks to its effective charge carrier management and light absorption properties. For instance, while some organic-based heterojunctions might show high conversion efficiencies at lower illumination levels, the **ATEHPQ** structure's ability to sustain high fill factors and conversion efficiencies across a range of illumination intensities highlights its robustness and adaptability. This performance suggests that the **ATEHPQ**-based heterojunction could be a strong candidate for applications requiring high efficiency and reliability in variable lighting conditions, making it comparable to or even exceeding the performance of other organic-based heterojunctions in certain respects [61,62].

## 4 Conclusions

In conclusion, this study successfully synthesized and characterized a novel thiadiazolylpyrano[3,2-*c*]quinoline derivative, **ATEHPQ**, with important implications for optoelectronic and photovoltaic applications. Quantum chemical calculations were used to analyze the compound's structural and electronic properties, which were experimentally confirmed using NMR spectroscopy and molecular electrostatic potential analysis. The compound showed promising nonlinear optical properties, outperforming urea, and demonstrated favorable drug-like characteristics based on Lipinski's and Veber's rules. Optical analysis revealed a direct energy bandgap of 2.43 eV, along with distinct absorption features, indicating its suitability for optoelectronic devices. Furthermore, the **ATEHPQ**-based heterojunction solar cell exhibited enhanced photovoltaic performance, achieving a power conversion efficiency of 4.64 % under 100 mW/cm^2^ illumination, significantly surpassing that of a bare silicon substrate. These results highlight **ATEHPQ**'s potential as a multifunctional material for use in advanced energy and optoelectronic technologies.

## CRediT authorship contribution statement

**Ibtisam Alali:** Writing – original draft, Funding acquisition, Data curation.

## Esthetical statement

This manuscript describes the original work and is not under consideration by any other journal. All authors approved the manuscript and this submission.”

Thank you for receiving our manuscript and considering it for review. We appreciate your time and look forward to your response.

## Funding

This work was funded by the Deanship of Graduate Studies and Scientific Research at Jouf University under grant No. (DGSSR-2024-02-01085).

## Declaration of competing interest

The authors declare that they have no known competing financial interests or personal relationships that could have appeared to influence the work reported in this paper.

## References

[1] Nalla S., Pavani Y., Ravi kumar G., Sumalatha P., Syed T., Subbarao M. (2024). Design, synthesis, anticancer evaluation, and molecular docking studies of 1,3,4-thiadiazole bearing 1,3,5-triazine-thiazoles. Synth. Commun..

[2] Demiraran S., Osmaniye D., Kaplancıklı Y.Z.A., Koçyigit-Kaymakçıoglu B., Tok F. (2024). Synthesis, characterization, biological evaluation and *in silico* studies of novel 1,3,4-thiadiazole derivatives as aromatase inhibitors. J. Mol. Struct..

[3] Zheng H., Kuang J., Zhang H., Niu X., Wu Z. (2022). Design, synthesis, and bioassay of novel 1-(3-chloropyridin-2-yl)-5-amino-4-pyrazole derivatives containing a 1,3,4-thiadiazole thioether or sulfone moiety, J Heterocycl. Chem.

[4] Kumar H., Dhameja M., Kurella S., Uma A., Gupta P. (2023). Synthesis, *in-vitro* α-glucosidase inhibition and molecular docking studies of 1,3,4-thiadiazole-5,6-diphenyl-1,2,4-triazine hybrids: potential leads in the search of new antidiabetic drugs. J. Mol. Struct..

[5] Upadhyay D.B., Vala R.M., Patel S.G., Patel P.J., Chi C., Patel H.M. (2023). Water mediated TBAB catalyzed synthesis of spiro-indoline-pyrano[3,2-*c*]quinolines as α-amylase inhibitor and in silico studies. J. Mol. Struct..

[6] Ramadan M., Elshaier Y.A.M.M., Aly A.A., Abdel-Aziz M., Fathy H.M., Brown A.B., Pridgen J.R., Dalby K.N., Kaoud T.S. (2021). Development of 2′-aminospiro [pyrano[3,2–*c*]quinoline]-3′-carbonitrile derivatives as non-ATP competitive Src kinase inhibitors that suppress breast cancer cell migration and proliferation. Bioorg. Chem..

[7] Ibrahim M.A., Emara A.A.A., Taha A., Adly O.M.I., Nabeel A.I., Aziz M.A., Salah N. (2023). Synthesis, characterization, TD-DFT, molecular docking, biological applications, and solvatochromic studies of some new metal complexes derived from semicarbazone of pyrano[3,2-*c*]quinoline-3-carboxaldehyde. Appl. Organomet. Chem..

[8] Abdel Halim S., Badran A., Roushdy N., Ahmed E.M., Ibrahim M.A., Farag A.A.M. (2023). A new hybrid structure based Pyranoquinoline-Pyridine derivative: synthesis, optical properties, theoretical analysis, and photodiode applications. J. Mol. Struct..

[9] Salah N., Taha A., Emara A.A.A., Adly O.M.I., Nabeel A.I., Aziz M.A., Ibrahim M.A. (2022). Novel NO_2_ semicarbazone ligand and its metal complexes as VEGFR-2 inhibitors: synthesis, spectral characterization, DFT calculations, molecular docking, antimicrobial and antitumor evaluation. Appl. Organomet. Chem..

[10] Zhong W.-Y., Song L.-Q., Zhou B., Zhou H.-T., Sun H.-S., Zhang H., Han J. (2023). Synthesis, mesomorphic and fluorescent properties of 1,3,4-oxadiazoles/thiadiazoles with a terminal 3-fluoro-4-cyanophenyl group. Tetrahedron.

[11] Zhu H., Zhang J., Zhang H., Bai J., Peng J., Jia J. (2024). Triphenylamine-based 1,3,4-thiadiazole derivative: solvatochromism, aggregation-induced emission and reversible turn-on mechanofluorochromism. J. Luminescence.

[12] Kohal R.F.B., Tarahhomi A. (2021). Evaluation of structural, spectroscopic, bonding and electronic properties of some organotin(IV)-phosphoric triamide complexes by using help of DFT, QTAIM and Hirshfeld surface investigations. Comput. Theor. Chem..

[13] Naseh M., Sedaghat T., Tarassoli A., Shakerzadeh E. (2013). DFT studies of ONO Schiff bases, their anions and diorganotin(IV) complexes: tautomerism, NBO and AIM analysis. Comput. Theor. Chem..

[14] Arora R., Issar U., Kakkar R. (2018). Theoretical investigation of organotin(IV) complexes of substituted benzohydroxamic acids. Comput. Theor. Chem..

[15] Badran A., Ahmed A., Nabeel A.I., Ibrahim M.A. (2024). Ring opening ring closure reactions with 5,9-diethyl-7-(chromon-3-yl)-7-hydroquinolino[3′,4′:5,6]pyrano[3,2-c] quinoline-6,8(5H,9H)-dione with some 1,2-binucleophiles: synthesis, characterization, DFT study and biological activity. J. Mol. Struct..

[16] Ahmed A., Ibrahim M.A., Badran A. (2023). Nucleophilic reactions with the novel 7-(chromon-3-yl)quinolino[3′,4′:5,6]pyrano[3,2-c]quinoline: synthesis, biological and computational studies. J. Mol. Struct..

[17] Chandini K.M., Lohith T.N., Shamanth S., Sridhar M.A., Mantelingu K., Lokanath N.K. (2023). Synthesis, structure elucidation, energy frameworks, and DFT calculations of 2,5-diphenyl-1,3,4-thiadiazole. J. Mol. Struct..

[18] Becke A.D. (1993). Density‐functional thermochemistry. III. The role of exact exchange. J. Chem. Phys..

[19] Zhang I.Y., Xu X. (2021). Exploring the limits of the XYG3-type doubly hybrid approximations for the main-group chemistry: the xDH@B3LYP model. J. Phys. Chem. Lett..

[20] Nakata M., Maeda T. (2023). PubChemQC B3LYP/6-31g∗//PM6 data set: the electronic structures of 86 million molecules using B3LYP/6-31G∗ calculations. J. Chem. Inf. Model..

[21] Moret M., Angona I.P., Cotos L., Yan S., Atz K., Brunner C., Baumgartner M., Grisoni F., Schneider G. (2023). Leveraging molecular structure and bioactivity with chemical language models for de novo drug design. Nat. Commun..

[22] Frisch M.J., Trucks G.W., Schlegel H.B., Scuseria G.E., Robb M.A., Cheeseman J.R., Scalmani G., Barone V., Mennucci B., Petersson G.A., Nakatsuji H., Caricato M., Li X., Hratchian H.P., Izmaylov A.F., Bloino J., Zheng G., Sonnenberg J.L., Hada M., Ehara M., Toyota K., Fukuda R., Hasegawa J., Ishida M., Nakajima T., Honda Y., Kitao O., Nakai H., Vreven T., Montgomery J.A., Peralta J.E., Ogliaro F., Bearpark M.J., Heyd J., Brothers E.N., Kudin K.N., Staroverov V.N., Kobayashi R., Normand J., Raghavachari K., Rendell A.P., Burant J.C., Iyengar S.S., Tomasi J., Cossi M., Rega N., Millam N.J., Klene M., Knox J.E., Cross J.B., Bakken V., Adamo C., Jaramillo J., Gomperts R., Stratmann R.E., Yazyev O., Austin A.J., Cammi R., Pomelli C., Ochterski J.W., Martin R.L., Morokuma K., Zakrzewski V.G., Voth G.A., Salvador P., Dannenberg J.J., Dapprich S., Daniels A.D., Farkas O., Foresman J.B., Ortiz J.V., Cioslowski J., Fox D.J. (2010).

[23] Sen P., Atmaca G.Y., Erdoğmuş A., Dege N., Genç H., Atalay Y., Yildiz S.Z. (2015). The synthesis, characterization, crystal structure and photophysical properties of a new meso-BODIPY substituted phthalonitrile. J. Fluoresc..

[24] Inac H., Ashfaq M., Dege N., Feizi-Dehnayebi M., Munawar K.S., Yağcı N.K., Çınar E.P., Tahir M.N. (2024). Synthesis, spectroscopic characterizations, single crystal XRD, supramolecular assembly inspection via hirshfeld surface analysis, and DFT study of a hydroxy functionalized schiff base Cu(II) complex. J. Mol. Struct..

[25] Tamer Ö., Dege N., Demirtaş G., Avcı D., Atalay Y., Macit M., Ağar A.A. (2014). An experimental and theoretical study on the novel (Z)-1-((naphthalen-2-ylamino) methylene) naphthalen-2 (1H)-one crystal. Spectrochim. Acta.

[26] Evecen M., Tanak H., Tinmaz F., Dege N., Ilhan I.O. (2016). Experimental (XRD, IR, and NMR) and theoretical investigations on 1-(2-nitrobenzoyl)3,5-bis(4-methoxyphenyl)-4,5-dihydro-1H-pyrazole. J. Mol. Struct..

[27] Ibrahim M.A., Hassanin H.M. (2019). Synthesis and reactions of the novel 6-ethyl-4-hydroxy-2,5-dioxo-5,6-dihydro-2H-pyrano[3,2-c]quinoline-3-carboxaldehyde. J. Heterocycl. Chem..

[28] Khalid M., Ali A., De la Torre A.F., Marrugo K.P., Concepcion O., Kamal G.M., Muhammad S., Al-Sehemi A.G. (2020). Facile synthesis, spectral (IR, mass, UV−Vis, NMR), linear and nonlinear investigation of the novel phosphonate compounds: a combined experimental and simulation study. Chem. Select.

[29] Khan M.U., Iqbal J., Khalid M., Hussain R., Braga A.A.C., Hussain M., Muhammad S. (2019). Designing triazatruxene-based donor materials with promising photovoltaic parameters for organic solar cells. RSC Adv..

[30] Aihara J. (1999). Reduced HOMO −LUMO gap as an index of kinetic stability for polycyclic aromatic hydrocarbons. J. Phys. Chem. A..

[31] Senet P. (1997). Chemical hardnesses of atoms and molecules from frontier orbitals. Chem. Phys. Lett..

[32] Milusheva M., Todorova M., Gledacheva V., Stefanova I., Feizi-Dehnayebi M., Pencheva M., Nedialkov P., Tumbarski Y., Yanakieva V., Tsoneva S., Nikolova S. (2023). Novel anthranilic acid hybrids— an alternative weapon against inflammatory diseases. Pharmaceut..

[33] Milusheva M., Gledacheva V., Stefanova I., Feizi-Dehnayebi M., Mihaylova R., Nedialkov P., Cherneva E., Tumbarski Y., Tsoneva S., Todorova M., Nikolova S. (2023). Synthesis, molecular docking, and biological evaluation of novel anthranilic acid hybrid and its diamides as antispasmodics. Int. J. Mol. Sci..

[34] Domingo L.R., Aurell M.J., Pérez P., Contreras R. (2002). Quantitative characterization of the global electrophilicity power of common diene/dienophile pairs in Diels–Alder reactions. Tetrahedron.

[35] Rezvani M., Darvish Ganji M., Jameh-Bozorghi S., Niazi A. (2018). DFT/TD-semiempirical study on the structural and electronic properties and absorption spectra of supramolecular fullerene- porphyrine-metalloporphyrine triads based dyesensitized solar cells. Spectrochim. Acta.

[36] Yamang H., Bhuyan J. (2024). Quest for iron(III) isoporphyrins: synthesis, characterization, reactivity and theoretical studies. J. Mole. Struct..

[37] Prabavathi N., Nilufer A., Krishnakumar V. (2012). Quantum mechanical study of the structure and spectroscopic (FT-IR, FT-Raman, ^13^C, ^1^H and UV), NBO and HOMO-LUMO analysis of 2-quinoxaline carboxylic acid. Spectrochim. Acta.

[38] Rahuman M.H., Muthu S., Raajaraman B.R., Raja M., Umamahesvari H. (2020). Investigations on 2-(4-Cyanophenylamino) acetic acid by FT-IR,FT-Raman, NMR and UV-Vis spectroscopy, DFT (NBO, HOMO-LUMO, MEP and Fukui function) and molecular docking studies. Heliyon.

[39] Faizan M., Afroz Z., Alam M.J., Rodrigues V.H., Ahmad S., Ahmad A. (2019). Structural, vibrational and electronic absorption characteristics of the monohydrate organic salt of 2-amino-5-bromo-6-methyl-4-pyrimidinol and 2,3-pyrazinedicarboxylic acid: a combined experimental and computational study. J. Mol. Struct..

[40] Kumar A., Deval V., Tandon P., Gupta A., D D’silva E. (2014). Experimental and theoretical (FTIR, FT-Raman, UV–vis, NMR) spectroscopic analysis and first order hyperpolarizability studies of non-linear optical material: (2E)-3-[4-(methylsulfanyl) phenyl]-1-(4-nitrophenyl) prop-2-en-1-one using density functional theory. Spectrochim. Acta.

[41] Shanmugavadivu T., Senthilkumar K., Dhandapani M., Muthuraja P., Balachandar S., Raman M.S. (2017). Theoretical and experimental evaluation of a new organic proton transfer crystal aminoguanidinium p-nitrobenzoate monohydrate for optical limiting applications. J. Phys. Chem. Solids..

[42] El Kalai F., Çınar E.B., Lai C.-Hung, Daoui S., Chelfi T., Allali M., Dege N., Karrouchi K., Benchat N. (2021). Synthesis, spectroscopy, crystal structure, TGA/DTA study, DFT and molecular docking investigations of (*E*)-4-(4-methylbenzyl)-6-styrylpyridazin-3(2*H*)-one. J. Mol. Struct..

[43] Ravi S., Sreedharan R., Raghi K.R., Manoj Kumar T.K., Naseema K. (2021). Experimental and theoretical studies on various linear and non-linear optical properties of 3-aminopyridinium 3,5-dinitrobenzoate for photonic applications. Braz. J. Phys..

[44] Lipinski C.A. (2004). Lead- and drug-like compounds: the rule-of-five revolution. Drug Discov. Today Technol..

[45] Fathima S.S.A., Meeran M.M.S., Nagarajan E.R. (2019). Synthesis of novel (E)-2-((anthracen-9-ylmethylene)amino)pyridin-3-ol and its transition metal complexes: multispectral characterization, biological evaluation and computational studies. J. Mol. Liq..

[46] Veber D.F., Johnson S.R., Cheng H.Y., Smith B.R., Ward K.W., Kopple K.D. (2002). Molecular properties that influence the oral bioavailability of drug candidates. J. Med. Chem..

[47] Lipinski C.A., Lombardo F., Dominy B.W., Feeney P.J. (2001). Experimental and computational approaches to estimate solubility and permeability in drug discovery and development settings. Adv. Drug Deliv. Rev..

[48] Tayeb H.O., Yang H.D., Price B.H., Tarazi F.I. (2012). Pharmacotherapies for Alzheimer's disease: beyond cholinesterase inhibitors. Pharmacol. The..

[49] Shityakov S., Neuhaus W., Dandekar T., Förster C. (2013). Analysing molecular polar surface descriptors to predict blood-brain barrier permeation. Int. J. Comput. Biol. Drug Des..

[50] Samavati Z., Samavati A., Ismail A.F., Awang A. (2022). Enhancement of organic sola cell efficiency by altering the zinc oxide photoanode nanostructure morphology. J. Nanostruct. Chem..

[51] Öztürk N., Bekmez M.G., Arslan B.S., Bulut E., Avcı D., Şişman İ., Nebioğlu M. (2024). Acridine-based metal-free organic dyes with various auxiliary acceptors for dye-sensitized solar cells. Dyes Pig.

[52] Bodur M.C., Duman S., Orak I., Saritas S., Baris O. (2023). The photovoltaic and photodiode properties of Au/Carmine/n-Si/Ag diode. Optics Laser Tech.

[53] Yang Y., Li X., Zhang Y., Zhang L., Zang L., Xu Z., Sun L. (2024). Synthesis of flower-like covalent organic frameworks for photocatalytic selective oxidation of sulfide. J. Taiwan Inst. Chem. Eng..

[54] Zhu Y., Lao J., Xu F., Sun L., Shao Q., Luo Y., Fang S., Chen Y., Yu C., Zou Y. (2024). A structurally controllable flower-shaped phosphide derived from metal-organic frameworks for high-performance supercapacitors. J. Electroanal. Chem..

[55] Zhang K., Shao Y., Jiang Y., Zhang L., Zhang S., Wang Y., Hu S., Xiang J., Hu X. (2024). Flower-like Cu/bone biochar for hydrogenation of vanillin: importance of evolution of organic matter in chicken bone on property of the catalysts. Mol. Catal..

[56] Hrostea L., Leontie L., Dobromir M., Doroftei C., Girtan M. (2020). On the electrical and optical properties stability of P3HT thin films sensitized with nitromethane ferric chloride solutions. Coatings.

[57] Pang C., Wu Y., Zhu J., Qin B., Ruan J. (2024). The study of adsorption mechanisms and kinetics of recovering Ag and Al on the bioleaching of end-of-life crystalline silicon photovoltaic cells. Sep. Purif. Technol..

[58] Ibrahim M.A., Badran A.-S., Abdel Halim S., Roushdy N., Atta A.A., Farag A.A.M. (2024). Examination of structural and spectrophotometric optical characteristics of nano-like flower quinolinyl carbonyl pyrazole-1-carbodithioate films: a new trend for optoelectronic applications. J. Mol. Struct..

[59] Farag A.A.M., Terra F.S., Mahmoud G.M. (2010). Structure, DC and AC conductivity of oxazine thin films prepared by thermal evaporation technique. Synth. Met..

[60] Sahin C., Mutlu D., Erdem A., Kilincarslan R., Arslan S. (2024). New cyclometalated iridium (III) complexes bearing substituted 2-(1H-benzimidazol-2-yl)quinoline: synthesis, characterization, electrochemical and anticancer studies. Bioorg. Chem..

[61] El-Ghamaz I.M., Gomha N.A., Emam H.M., Altowyan A.S. (2023). Synthesis, spectral, and theoretical characterization of new azo dye derivatives. J. Mol. Struct..

[62] Almotiri R.A., Alkhamisi M.M., Wassel A.R., El-Mahalawy A.M. (2022). Optical dispersion and photovoltaic performance of safranin thin films solar cells in hybrid organic-inorganic isotype heterojunction configuration. Mater. Res. Bull..

