# Supplementary material for: Synthesis, spectral analysis, and DFT studies of the novel pyrano[3,2-c] quinoline-based 1,3,4-thiadiazole for enhanced solar cell performance
Source: Heliyon. 2024 Oct 17;10(20):e39468. doi: 10.1016/j.heliyon.2024.e39468 (PMC11533592; doi:10.1016/j.heliyon.2024.e39468)
Supplement: Multimedia component 1 [file mmc1.docx]

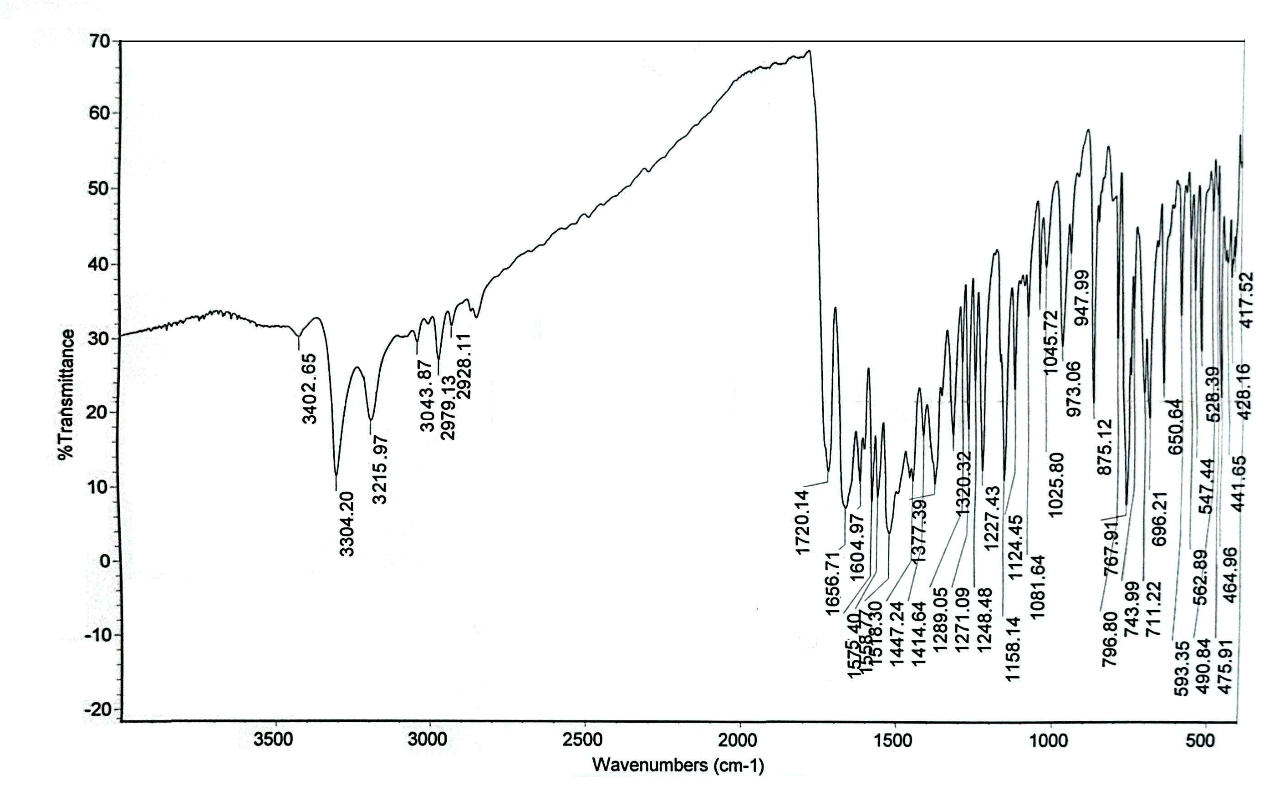


**Figure S1:** IR spectrum of compound **3**


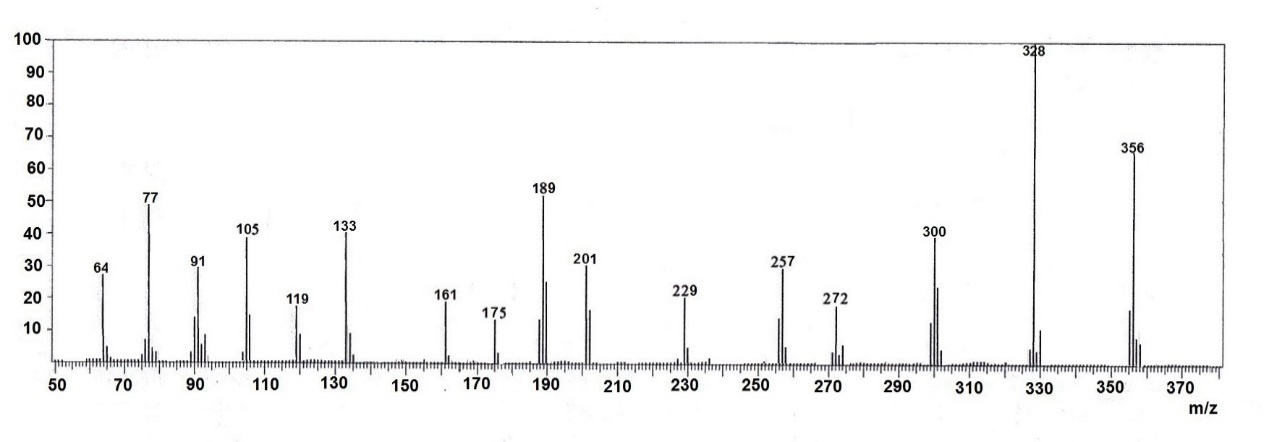


**Figure S2:** Mass spectrum of compound **3**


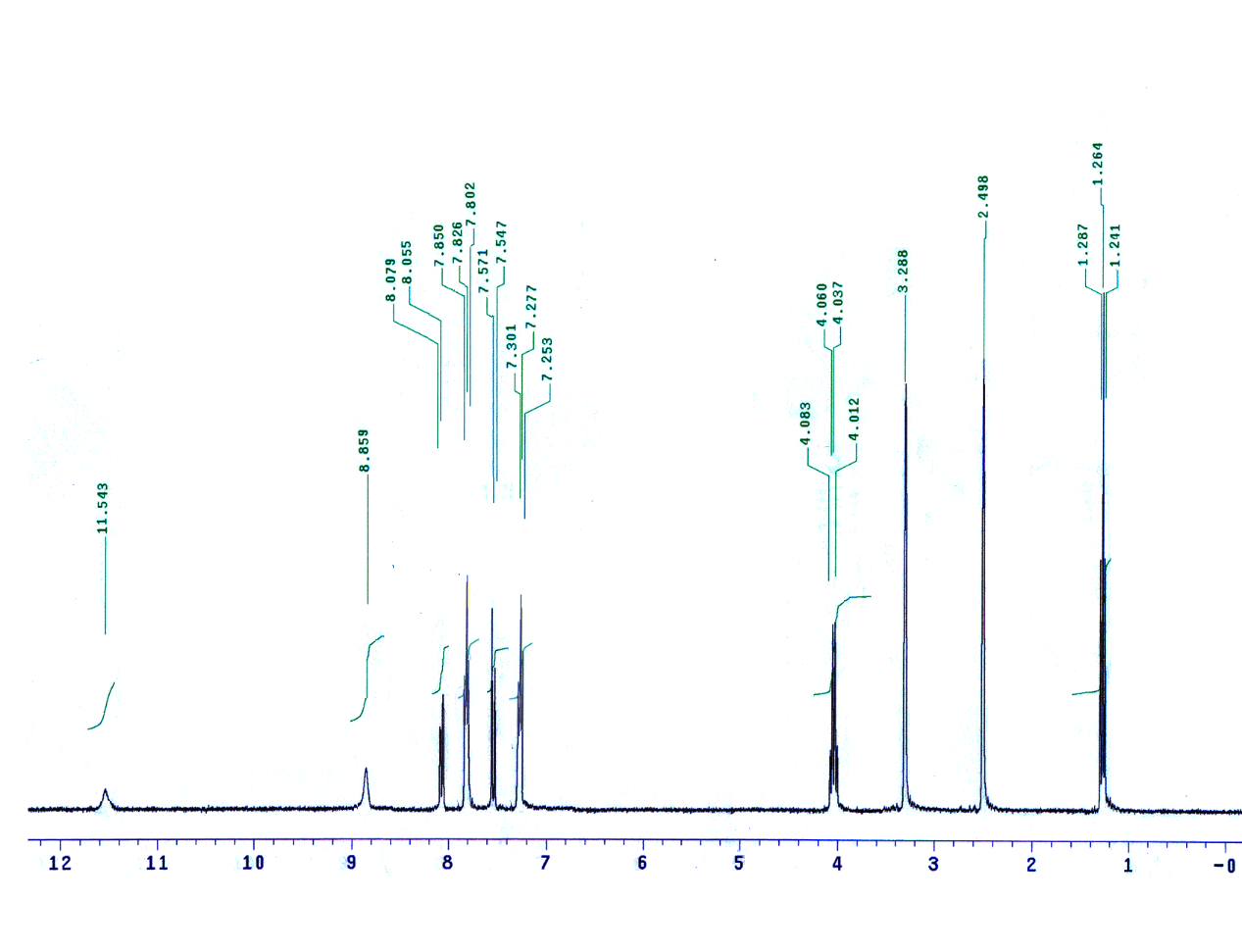

**Figure S3:** (a) Experimental and (b) Calculated ^1^H NMR spectra of compound **3** at B3LYP/6-311++G(d,p).


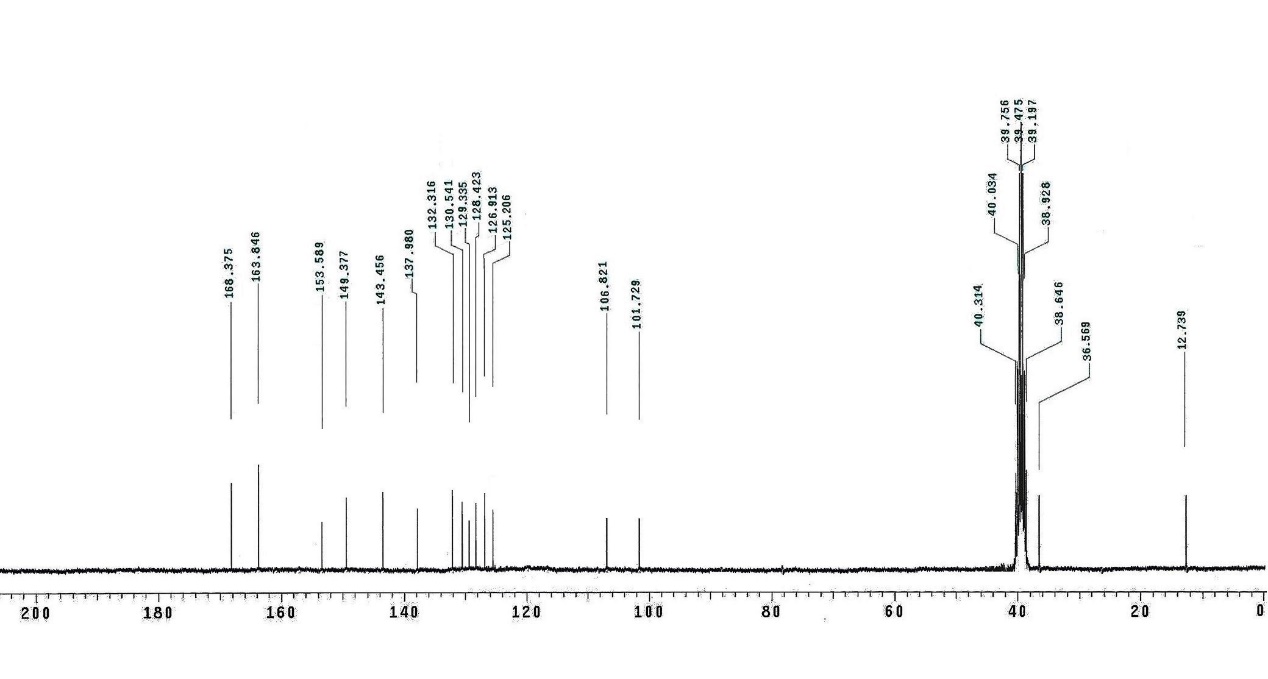

**Figure S4:** (a) Experimental and (b) Calculated ^13^C NMR spectra of compound **3** at B3LYP/6-311++G(d,p).
